# Mitochondrial 16S rRNA Is Methylated by tRNA Methyltransferase TRMT61B in All Vertebrates

**DOI:** 10.1371/journal.pbio.1002557

**Published:** 2016-09-15

**Authors:** Dan Bar-Yaacov, Idan Frumkin, Yuka Yashiro, Takeshi Chujo, Yuma Ishigami, Yonatan Chemla, Amit Blumberg, Orr Schlesinger, Philipp Bieri, Basil Greber, Nenad Ban, Raz Zarivach, Lital Alfonta, Yitzhak Pilpel, Tsutomu Suzuki, Dan Mishmar

**Affiliations:** 1 Department of Life Sciences, Ben-Gurion University of the Negev, Beer Sheva, Israel; 2 Department of Molecular Genetics, the Weizmann Institute of Science, Rehovot, Israel; 3 Department of Chemistry and Biotechnology, University of Tokyo, Tokyo, Japan; 4 The Ilse Katz Institute for Nanoscale Science and Technology, Beer Sheva, Israel; 5 Department of Biology, Institute of Molecular Biology and Biophysics, Zurich, Switzerland; University of Maryland, UNITED STATES

## Abstract

The mitochondrial ribosome, which translates all mitochondrial DNA (mtDNA)-encoded proteins, should be tightly regulated pre- and post-transcriptionally. Recently, we found RNA-DNA differences (RDDs) at human mitochondrial 16S (large) rRNA position 947 that were indicative of post-transcriptional modification. Here, we show that these 16S rRNA RDDs result from a 1-methyladenosine (m^1^A) modification introduced by TRMT61B, thus being the first vertebrate methyltransferase that modifies both tRNA and rRNAs. m^1^A947 is conserved in humans and all vertebrates having adenine at the corresponding mtDNA position (90% of vertebrates). However, this mtDNA base is a thymine in 10% of the vertebrates and a guanine in the 23S rRNA of 95% of bacteria, suggesting alternative evolutionary solutions. m^1^A, uridine, or guanine may stabilize the local structure of mitochondrial and bacterial ribosomes. Experimental assessment of genome-edited *Escherichia coli* showed that unmodified adenine caused impaired protein synthesis and growth. Our findings revealed a conserved mechanism of rRNA modification that has been selected instead of DNA mutations to enable proper mitochondrial ribosome function.

## Abstract 2

Two solutions were selected during evolution to allow proper function of the vertebrate mitochondrial 16S ribosomal RNAeither RNA methylation by a tRNA methyltransferase or ancient evolutionary mutation.

## Author Summary

RNA modifications constitute an important layer of information, with functional implications that are not written in the underlying DNA sequence. Recently, we observed an apparent RNA-DNA difference (RDD) at position 947 of the human mitochondrial 16S ribosomal RNA (rRNA), but its nature and mechanism were unclear. Here we show that this disparity reflects an m^1^A modification (methylation at position 1 of the adenine moiety), and demonstrated by a combination of knock-down experiments in cells and in vitro methylation assays that the tRNA methyltransferase TRMT61B is the best candidate enzyme to introduce this modification. We also show that this modification is present in most of the 16S rRNA molecules in isolated mitochondrial ribosomes, and that it occurs in all vertebrates with an adenine (90% of the vertebrates), but not in those with a thymidine at this 16S rRNA position. Finally, as the first step towards understanding the functional importance of this rRNA modification, we used a genome-edited bacterial system to demonstrate that an unmodified adenine reduced the growth and translation rates of the bacteria as compared to both wild-type bacteria and mutant bacteria with a thymidine in the relevant position. Hence, three solutions were selected during evolution to allow proper function of the mitochondrial 16S rRNA—either RNA modification or two alternative ancient evolutionary DNA mutations.

## Introduction

Most RNAs are enzymatically modified after transcription. To date, more than 100 different species of chemical modifications have been identified in various RNA molecules from all domains of life [1]. Historically, analyses of RNA modifications have been limited to abundant RNA molecules such as tRNA, rRNA, or UsnRNA. Recently, transcriptome-wide analyses using deep-sequencing combined with biochemical approaches have facilitated the identification of several modified bases in mRNAs and non-coding RNAs [2–5]. Additionally, transcriptomes were compared to their corresponding genomic sequences as a practical approach for the detection of RNA-DNA differences (RDDs) and identification of the canonical A-to-I and C-to-U RNA editing sites in diverse eukaryotes [6]. Accumulating evidence suggests the presence of non-canonical RDD sites (i.e., edits other than A-to-I [G] or C-to-U) [7–9]. However, their very existence and general importance has been questioned [10], and neither the mechanisms generating non-canonical RDDs nor conclusive experimental evidence for their functional role have been described in vertebrates.

It was previously suggested that many non-canonical RDDs are not editing events per-se but rather reflect RNA modifications [8]. RNA modifications, such as methylation of specific bases, occur in mitochondrial RNAs in many vertebrates and invertebrates [11–14]. These modifications play crucial roles in mitochondrial activity, and their absence leads to pathological consequences [15]. While RNA modifications in mitochondrial tRNAs of mammals were thoroughly mapped [16], the mitochondrial ribosomal (r)RNAs have been far less investigated.

By sequencing both the genome and corresponding transcriptome of the human mitochondria in lymphoblastoid cells, we recently identified three new RDD sites [8] that were also corroborated in a larger human sample size [13]. The most prevalent RDD occurred at adenine 947 of human mitochondrial 16S rRNA (mitochondrial DNA [mtDNA] position 2617). Specifically, we showed that this position was a mixture of A, T, or G in RNA-seq reads, suggesting the presence of an unidentified modified base [8].

Recently, cryo-EM structures of mammalian mitochondrial ribosomes (mitoribosomes) have been solved at high resolution, sufficient to map residues of rRNAs [17,18]. A947 is located in helix 71 (H71) of 16S rRNA in close proximity to the inter-subunit bridge B3, suggesting that the modified base at this position may play a functional role in mitochondrial translation. In this study, we investigated the function of A947 and its RDD. We found that the non-canonical mitochondrial 16S rRNA RDDs resulted from a 1-methyladenosine (m^1^A) RNA modification and identified the candidate modifying enzyme. We demonstrate that the modification occurred in most vertebrates and is enriched in the mature mammalian mitoribosome. Finally, we used a bacterial model to directly link the nucleotide identity of 16S rRNA position 947 to cellular growth and protein translation. Indeed, mutations in the equivalent structural position of A947 in *Escherichia coli* imply functional importance for modifying this base during evolution.

## Results

### Identification of Methyladenosine at Position 947 in Human Mitochondrial 16S rRNA

We previously hypothesized that the observed 16S rRNA RDDs in RNA-seq represent an RNA modification that was manifested as a mixture of reads with thymine, adenine, and lower occurrences of guanine. However, the nature of this putative modification and its underlying mechanism remained to be elucidated. Previous RNA-seq analysis of templates harboring a m^1^A modification [19] resulted in nucleotide distribution of sequencing reads similar to our observed reads in the mitochondrial 16S rRNA transcript at position 947 [8].

**Fig 1:**
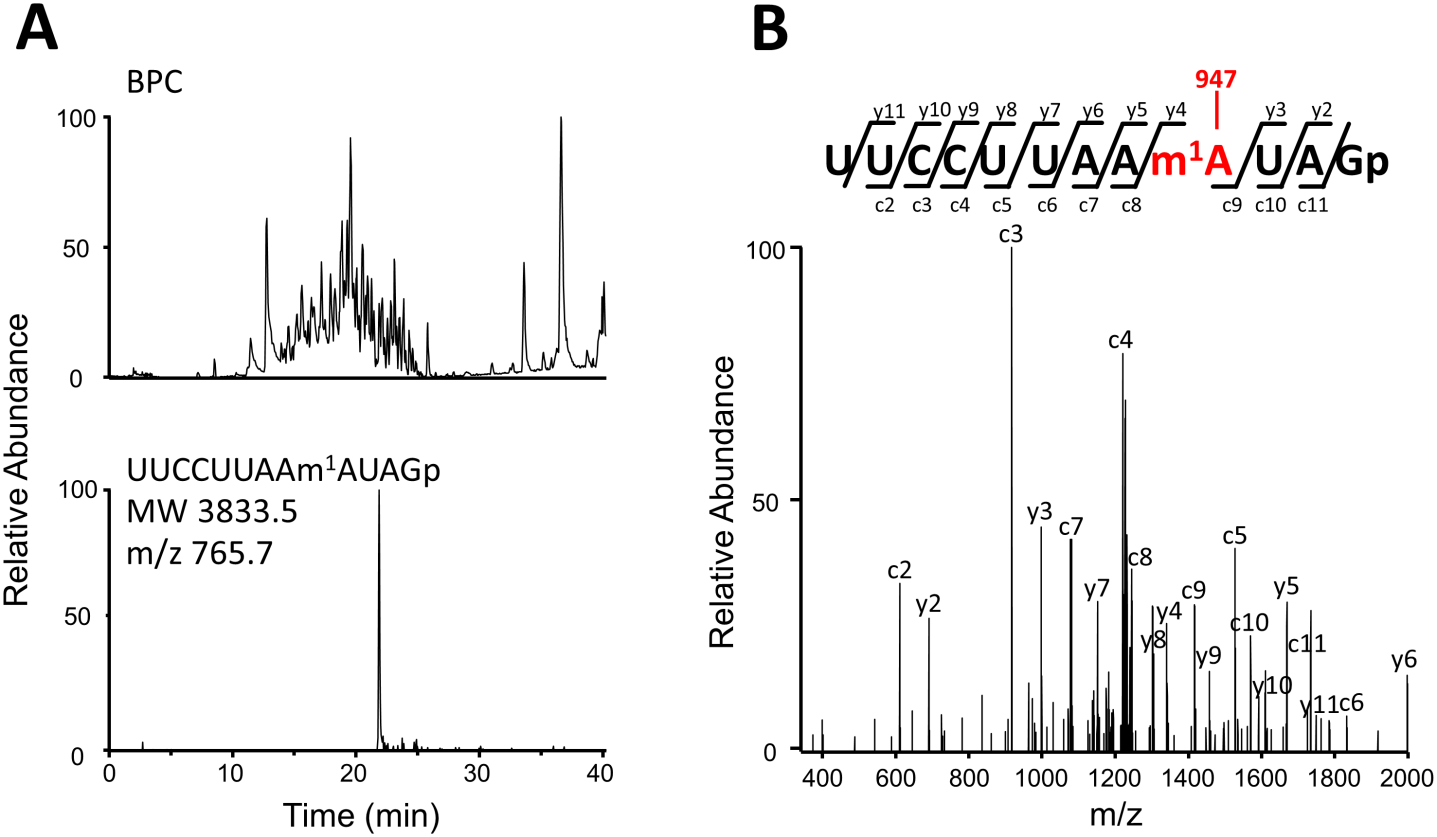
Human mitochondrial 16S rRNA is methylated at position 947. (**A**) Capillary LC/ESI-MS analysis of RNA fragments of human mitochondrial 16S rRNA digested with RNase T_1_. The upper panel shows a base-peak chromatogram (BPC), and the lower panel represents mass chromatogram for detecting quintuple (-5)-charged ion of the methylated 12-mer fragment (UUCCUUAAm^1^AUAGp, m/z 765.7). (**B**) Collison-induced dissociation spectrum of the methylated 12-mer fragment. The sequence was confirmed by assignment of the product ions. Nomenclature for the product ions is in accordance with a previous report [22].

To elucidate the chemical identity of 16S rRNA position 947, we isolated 16S rRNA from HeLa cells and analyzed its modifications by capillary liquid chromatography and nano electrospray mass spectrometry [20–22]. By assigning RNase T_1_-digested fragments of 16S rRNA, we observed known 2’-*O* methylations including Gm1145, Um1369 and Gm1370 (S1 Fig). In addition, we clearly detected the mono-methylated RNA fragment (positions 939–950, MW 3833.5) containing the RDD site at position 947 (Fig 1A). Further probing of the RNA fragment by collision-induced dissociation revealed that the methylation occurs at the adenosine residue occupying position 947 (Fig 1B).

### TRMT61B Introduces 1-Methyladenosine at Position 947 of Human Mitochondrial 16S rRNA

It is known that two isozymes introduce the m^1^A modification in human mitochondrial tRNAs [16]. TRMT10C is a subunit of the mitochondrial RNase P complex that additionally acts as a methyltransferase generating m^1^A as well as m^1^G at position 9 in mitochondrial tRNAs [23]. TRMT61B, another methyltransferase, is responsible for m^1^A at position 58 in some mitochondrial tRNAs [24]. Notably, a recent Genome Wide Association Study revealed association between the levels of our observed 16S rRNA RDDs with SNPs in TRMT61B [13]. We thus hypothesized that the RDD at 16S rRNA position 947 echoes an m^1^A modification, likely introduced by TRMT61B.

**Fig 2:**
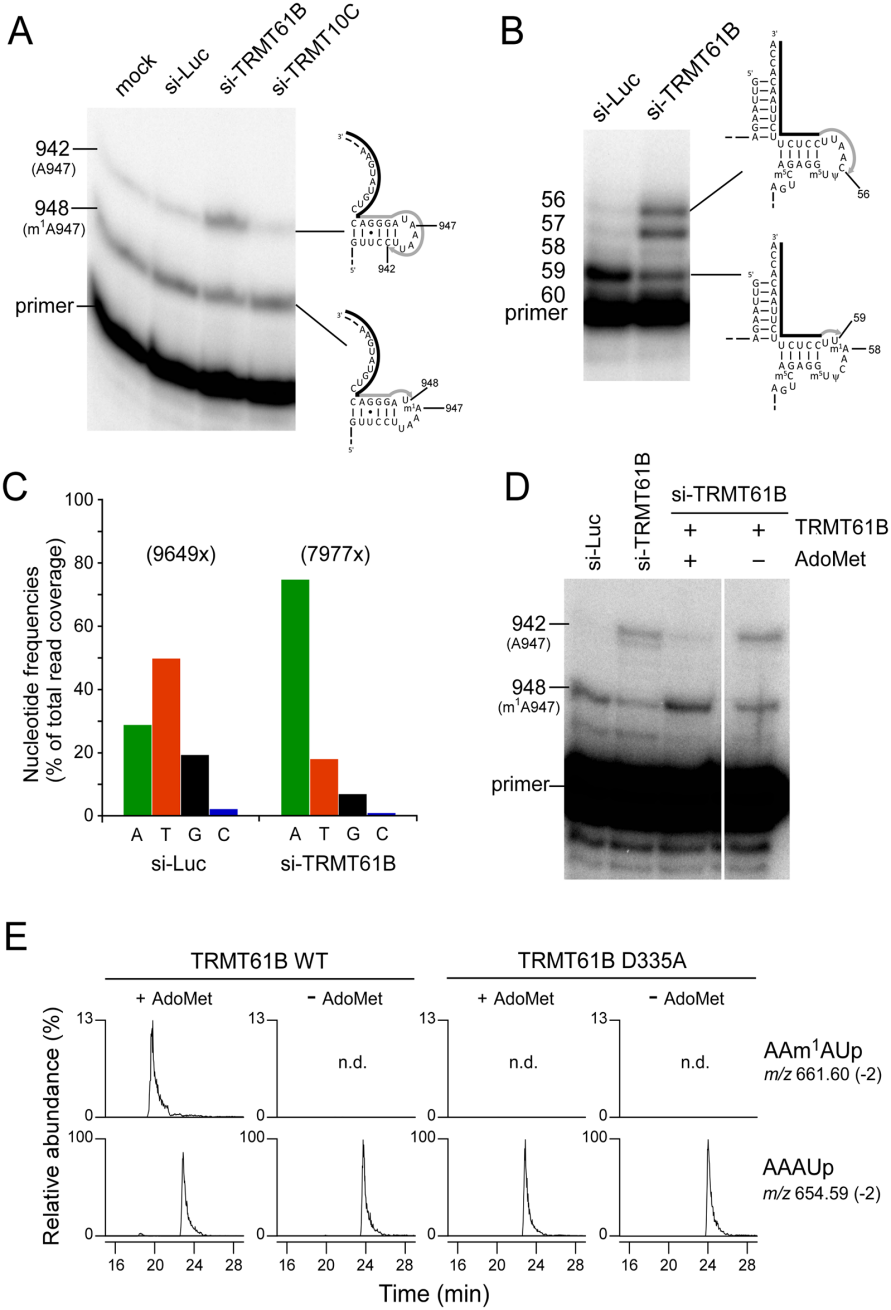
TRMT61B is responsible for m^1^A947 in mitochondrial 16S rRNA. (A) Detection of hypomodified m^1^A947 in mitochondrial 16S rRNA from siRNA-treated HeLa cells. After knockdown of luciferase (si-Luc, control), TRMT61B, or TRMT10C mRNAs, primer extension was used to detect methylated or non-methylated A947. The cells were transfected twice with siRNA and harvested 4 days after the first transfection. The knockdown efficiencies of TRMT61B and TRMT10C mRNAs were quantified by qRT-PCR and normalized to ACTB mRNA. The steady-state levels of both mRNAs were decreased to 5.7% compared to the mock cells. The primers are shown as solid lines next to the rRNA or tRNA, and nascent cDNAs synthesized from the primers are depicted as gray lines. (B) Detection of hypomodified m^1^A58 in mitochondrial tRNALeu^(UUR)^ to confirm TRMT61B knockdown. (C) Nucleotide frequencies in cDNA reads corresponding to position 947 in human mitochondrial 16S rRNA. RNA-seq reads of total RNAs from HeLa cells treated with siRNAs targeting luciferase (control) or TRMT61B were mapped against the human mtDNA sequence. Nucleotides frequencies (%) were calculated from the total read coverage. Exact values are available in S1 Data. (D) In vitro reconstitution of m^1^A947 with recombinant TRMT61B in the presence of AdoMet. Total RNAs from HeLa cells treated with siRNAs for TRMT61B (si-TRMT61B) were incubated with recombinant TRMT61B in the presence or absence of Ado-Met. m^1^A947 formation in mitochondrial 16S rRNA was detected by primer extension. Total RNAs of si-Luc and si-TRMT61B were used as controls for primer extension. (E) In vitro reconstitution of m^1^A947 with wild-type TRMT61B and its active-site D335A mutant. The 114-mer RNA segment including Helix 71 (G866-U979) of human mitochondrial 16S rRNA was incubated with wild-type TRMT61B or its D335A mutant in the presence or absence of Ado-Met, followed by RNase A digestion, and subjected to LC/MS analysis. Mass-spec chromatograms detect doubly-charged negative ions of the tetramer fragment carrying m^1^A947 (upper panels, positions 945–948, m/z 661.60, and MW 1325.21) and the corresponding unmodified fragment (lower panels, m/z 654.59, MW 1311.19).

To examine whether TRMT61B or TRMT10C catalyzes the formation of methyladenosine at position 947 in 16S rRNA, we knocked down each of them by siRNAs in HeLa cells, followed by total RNA extraction to obtain templates for primer extension. In control experiments of mock or luciferase knockdown (Fig 2A), the cDNA extended from the primer was strongly arrested at position 948, and partially extended to C942 by inserting dideoxy guanosine. This indicated that A947 is partially methylated. In addition, we also observed a clear band due to m^1^A58 in tRNA^Leu(UUR)^ from HeLa cells treated by luciferase siRNA (Fig 2B). Upon knockdown of TRMT61B (Fig 2A), nearly half of the cDNA extended past position 947 and stopped at position 942. This demonstrated that the methyladenosine at position 947 was m^1^A, introduced by TRMT61B. In contrast, knock down of TRMT10C did not lead to altered cDNA extension. As a positive control, hypomodification of m^1^A58 in tRNA^Leu(UUR)^ was observed when TRMT61B was repressed (Fig 2B). To confirm this observation by RDD, total RNAs from HeLa cells treated by siRNAs targeting for TRMT61B or luciferase as a control were subjected to RNA-seq analyses, followed by mapping to the mtDNA sequence (Fig 2C, S1 Data). Mixed nucleotide frequencies at position 947 observed in the cells treated with the control siRNA were dramatically altered and converged into adenosine in the cells treated with siTRMT61B, supporting the result from the primer extension experiment. Moreover, we observed a slight decrease in the read coverage around position 947. This observed pattern is in agreement with a recently published report describing the signature of m^1^A in RNA-seq data [25]. Notably, the observed levels of reads with either a T or a G decreased (as well as the reduction in coverage) in the RNA extracted from the siTRMT61B cells (Fig 2C).

To demonstrate that TRMT61B is a methyltransferase directly responsible for m^1^A947 formation in mitochondrial 16S rRNA, we carried out an in vitro reconstitution of m^1^A using recombinant TRMT61B. Total RNA extracted from HeLa cells treated by siRNA targeting TRMT61B was incubated with recombinant TRMT61B in the presence of AdoMet. m^1^A947 formation was specifically detected by primer extension assay (Fig 2D). In the presence of both recombinant TRMT61B and AdoMet, the cDNA band that extended up to position 942 decreased, and the cDNA band that arrested at position 948 clearly increased, indicating that m^1^A947 was reconstituted in vitro. In the negative control experiment, m^1^A947 was not introduced without AdoMet. Moreover, using in vitro transcription, we prepared a 114-nucleotide-long RNA segment (16S rRNA positions 866–979) and performed an in vitro methylation assay by TRMT61B. As a negative control, we prepared an active-site mutant of TRMT61B (D335A mutation), according to the biochemical study on TrmI [26], a bacterial ortholog of TRMT61B. Then, we carried out an in vitro methylation assay of the 114-mer RNA segment with either the wild-type TRMT61B or the D335A mutant in the presence or absence of AdoMet, followed by RNase A digestion, and subjected them to capillary LC/nano-ESI-MS analysis. The results indicate that TRMT61B clearly introduced the m^1^A947 in the 114-mer RNA segment in the presence of AdoMet (Fig 2E). Moreover, the methylated tetramer (AAm^1^AUp) produced by RNase A digestion was probed by CID, and its sequence was confirmed by assignment of the product ions (S2 Fig). As expected, the D335A TRMT61B mutant failed to introduce m^1^A947 in the segment (Fig 2E). Taken together, these results clearly demonstrate that the RDD site at 16S rRNA position 947 is m^1^A introduced by mitochondrial methyltransferase TRMT61B.

### m^1^A947 Likely Occurs in Mitochondrial 16S rRNA throughout Vertebrate Evolution

**Fig 3:**
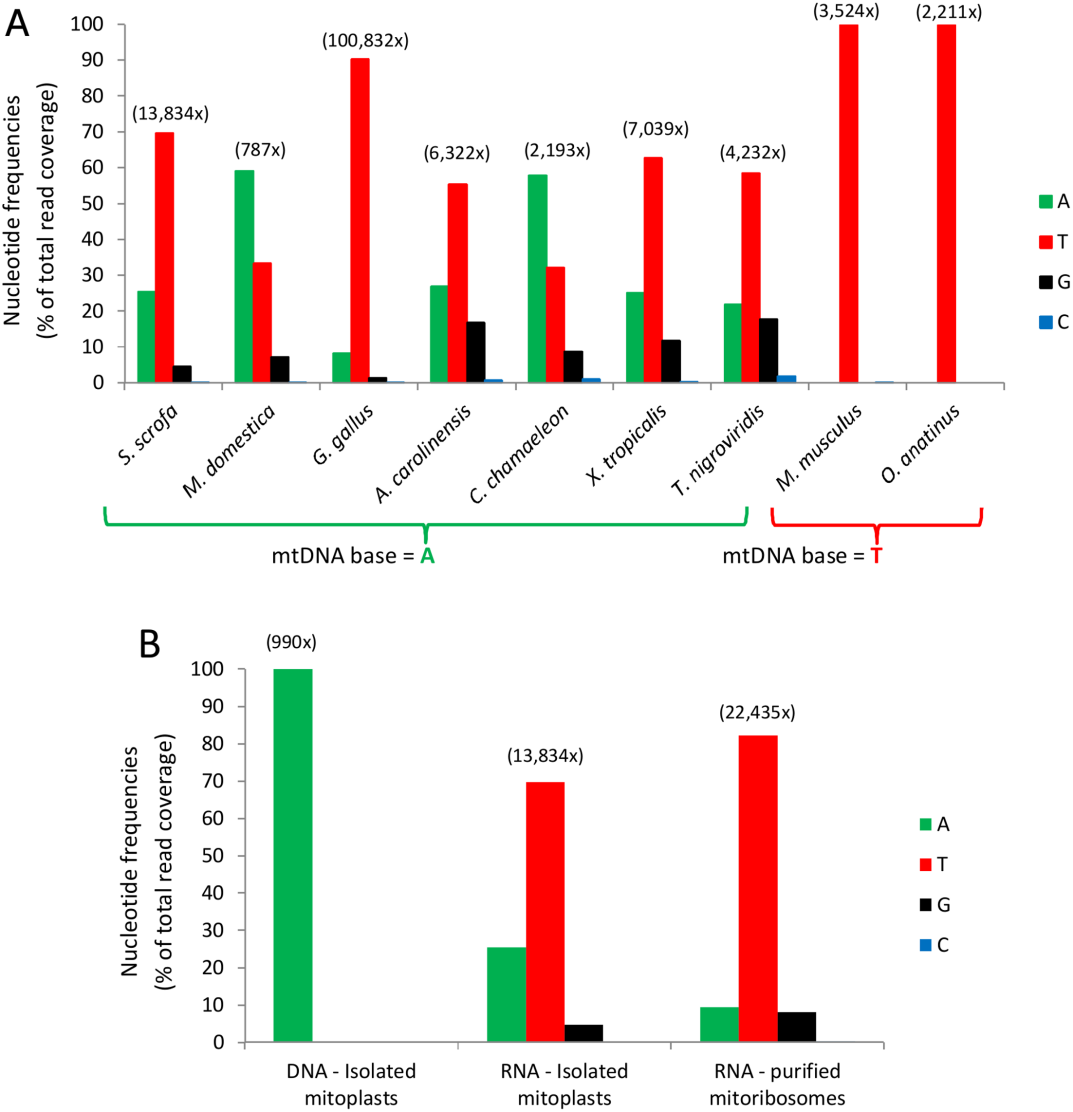
Nucleotide distribution in reads corresponding to 16S rRNA position 947 in humans. (**A**) RNA-Seq reads from nine species were mapped to their corresponding mtDNA sequence. Notice that species with an adenine in their mtDNA exhibit the RDDs, while species with a thymine do not (species 8–9). Total read coverage of all samples is shown above each species. (**B**) Coverage and nucleotide distribution of deep sequencing of *Sus scrofa* samples at the orthologue of human 16S rRNA position 947. The right histogram trio stem from an isolated ribosome (enriched for mature rRNA), and the middle trio stem from total mitochondrial RNA (which contains a mixture of mature and premature rRNAs). The left-most trio of histograms stem from mitochondrial DNA. Notice that the level of m^1^A modification increased in the right trio as compared to the middle one. Exact values are available in S1 Data.

Our previous phylogenetic analysis revealed high conservation of mtDNA position 2617, i.e., 16S rRNA position 947 [8]. Specifically, this position was an adenine in nearly 90% of all tested vertebrate species, while the remaining 10% had a thymine. The above findings are consistent with the idea that the presence of the m^1^A modification results in a mixture of reads with mostly adenine or thymine and less with a guanine in RNA-seq data, as previously observed [19]. To assess the extent to which position A947 is methylated across vertebrate phylogeny, we analyzed RNA-seq data from nine species representing major vertebrate taxa (placental mammals, marsupials, monotremes, birds, reptiles, amphibians, and bony fish) (Fig 3, S1 Data). Notably, special care was employed to assure proper RDD identification while excluding sequencing and mapping errors (see Materials and Methods) [8].

Our analysis revealed that RDDs (A-to-U and A-to-G) occurred in the 16S rRNA of all tested species in which an adenine occupied mtDNA position 2617, indicating the presence of m^1^A947 in these 16S rRNAs. In contrast, species with a thymine in their mtDNA maintained a uridine in their RNAs (Fig 3, S1 Data). Notably, the RDD levels varied among the tested species, possibly due to physiological differences between different vertebrates. These observations further support our interpretation that position 947 of 16S rRNA is modified and that this modification is highly conserved across vertebrates.

### m^1^A947 Is Highly Enriched in the Mature Mitoribosome

The m^1^A947 modification was identified in RNA-seq data generated from total RNA samples, harboring both mature and premature mitochondrial transcripts. As a first step to elucidate the functional importance of this modification, we assessed the extent to which 16S rRNA position 947 is present in the modified version in the mature ribosome. To this end, we purified whole mitochondria and isolated mitoribosomes from a single *Sus scrofa* liver specimen and sequenced mtDNA and RNA from the isolated mitochondria as well as RNA from the purified mitoribosome. As expected, 100% of mtDNA reads from isolated mitochondria showed an adenine at the *S*. *scrofa* orthologous position of human mtDNA position 2617 (Fig 3B, S1 Data). Remarkably, while RDDs appeared in ~75% of the mitochondrial total RNA sample, their prevalence increased to ~90% in the purified mitoribosome (*p* < 10E-10, χ2 test, Fig 3B, S1 Data). This RDD enrichment in the mammalian mitoribosome supports the interpretation that the mature mitoribosome likely almost entirely contains m^1^A947 16S rRNA, thus further supporting the functional importance of this modification.

### Protein Translation Is Impaired in Mutated Bacterial Ribosomes with Unmodified Adenine at the Orthologous Position of 16S rRNA Position 947

**Fig 4:**
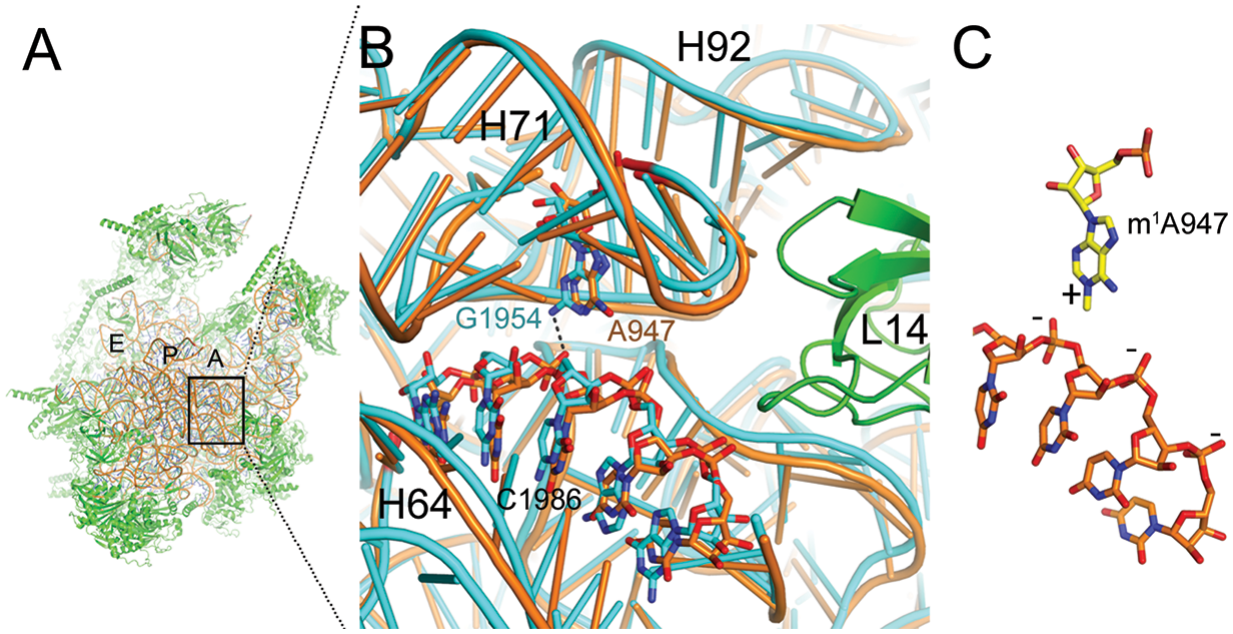
High structural conservation between *S*. *scrofa* mitoribosome and *E*. *coli* ribosome at position 947. (**A**) The structure of the porcine mitoribosomal large subunit (PDB accession code 4v1a and 4v19) shown from the subunit interface side. The ribosomal RNA is shown in brown and the ribosomal proteins in green. The ribosomal tRNA A-, P-, and E-binding sites are indicated. (**B**) Sticks-and-ribbon representation of interaction between helices H71 and H64 in *S*. *scrofa* (brown) mitoribosome or *E*. *coli* (turquoise) ribosome (PDB accession code 4ybb). The hydrogen bond that is likely disrupted by an adenine in position 947 is represented as a dashed line. (**C**) The positively charged m^1^A947 stabilizes the structure by interacting with the negatively charged H64 backbone. Numbers refer to the positions of *E*. *coli* ribosomal RNA.

Structural analysis of position 947 of 16S rRNA revealed that it is likely involved in anchoring H71 by forming interactions with H64 and H92 of the 39S subunit (Fig 4).

**Fig 5:**
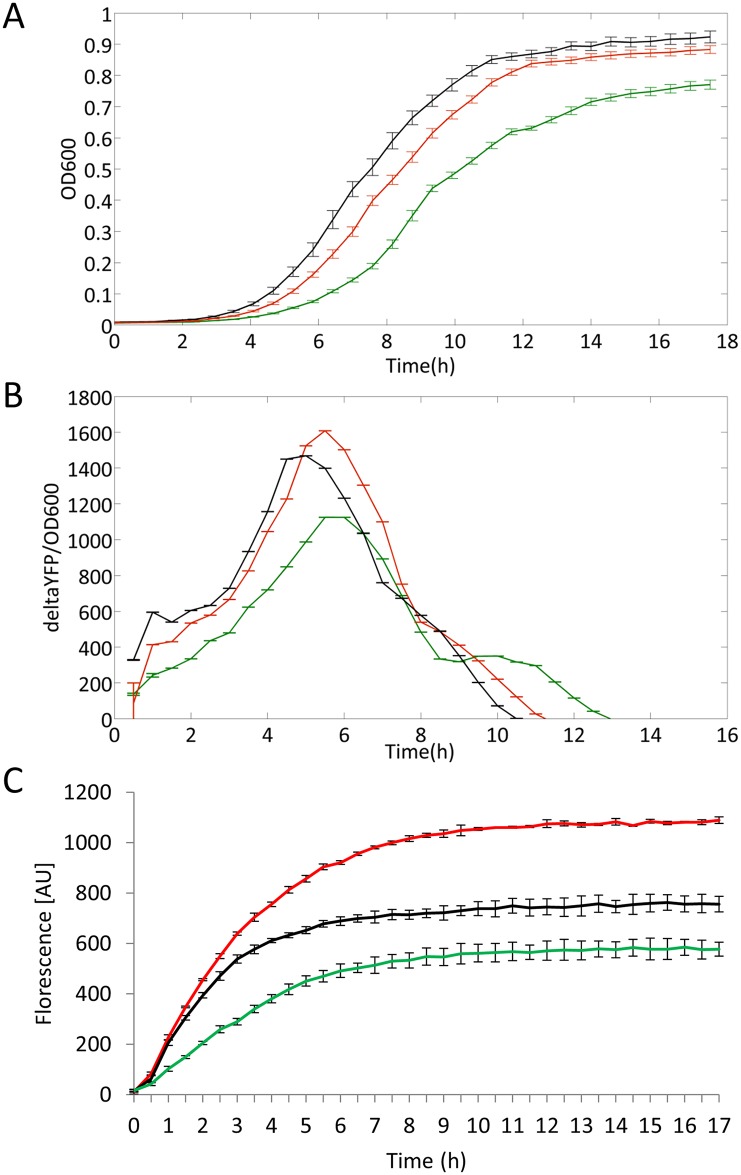
*E*. *coli* mutant strains harboring adenine at the orthologue of mitochondrial 16S rRNA position 947 showed impaired growth and protein synthesis. (**A**) Strains harboring a guanine (black line) or a thymine (red line) grew faster than strains harboring an adenine (green line). Lines represent an average with standard deviation of 42 repeated experiments. (**B**) *E*. *coli* harboring either a thymine (red line) or a guanine (black line) translated YFP more efficiently than *E*. *coli* strains harboring an adenine (green line). Lines represent average with standard deviations of 42 repeated experiments. (**C**) In vitro green fluorescent protein (GFP) synthesis of the different samples was measured over time by fluorescence excitation. Elevated GFP synthesis is observed in strains with ribosomes harboring a guanine (black line) or a thymine (red line) in comparison to strains harboring an adenine (green line). Lines represent average with standard deviation of three repeated experiments. Exact values are available in S1 Data. All experiments were repeated independently by using a second MAGE strain (one for adenine and one for thymine), which gave the same results.

Notably, this position is structurally conserved from bacteria to mammals in both mitochondrial and cytoplasmic ribosomal structures [27–30]. Our RNA-seq analysis of diverse vertebrate species showed an RDD in species with an adenine, but not with a thymine, at 16S rRNA position 947. Furthermore, this position is occupied by a thymine in the human cytoplasmic ribosome and by a guanine in 95% of all tested bacterial species. We therefore hypothesized that three functional alternatives arose during evolution, all of which are capable of maintaining full ribosomal activity: (A) an unmodified thymine (human cytoplasmic and 10% of the vertebrate mitochondrial ribosomes), (B) an unmodified guanine (in most bacteria), and (C) an m^1^A modification in 90% of the mitochondrial ribosomes. According to this hypothesis, forcing an unmodified adenine into the mature ribosome would interfere with its activity. Because there is no available technology to modify specific mtDNA nucleotides in cells, we chose *E*. *coli* as our model system to test this hypothesis. Using the genome engineering technology MAGE [31], we successfully replaced the endogenous bacterial nucleotide at position 1954, which corresponds to human mitochondrial 16S rRNA position 947, in all seven 23S rRNA gene copies. We independently replaced the wild-type guanine for either a thymine or an adenine (S3 Fig) and tested the resulting strains in terms of growth rate and translation efficiency. Remarkably, strains harboring an adenine grew significantly slower as compared to strains harboring either the WT base (guanine) or a thymine (Fig 5A, S1 Data). This result is consistent with our hypothesis that the presence of either thymine or guanine is important for cell growth, and that an unmodified adenine has a negative effect at this position on the mitochondrial and bacterial translational machineries.

Next, we aimed at assessing the effect of the mutations on ribosomal activity by examining the impact of each base at the modified position on protein synthesis. Indeed, in vivo examination of yellow fluorescent protein (YFP) production revealed that strains harboring an adenine at position 1,954 showed decreased maximal protein synthesis rate (64.4% and 76.2% in the mutant and WT, respectively) as well as a decrease in total protein production (75% and 83.6% in the mutant and WT, respectively; Fig 5B, S1 Data). This observation suggests that the major growth defect of the bacterial strain harboring an adenine at position 1,954 is caused by impaired protein synthesis. Interestingly, the strain with a thymine demonstrated a comparable, and even higher, maximal protein production rate (109% in the mutant as compared to WT) as well as total protein production (110.1% in the mutant, as compared to WT; Fig 5B, S1 Data). These results were further validated by a bacterial in vitro translation assay using cell-free synthesis of green fluorescent protein (GFP) as a reporter (Fig 5C, S1 Data). Taken together, our experiments strongly support our hypothesis that mitochondrial 16S rRNA position 947 should either be a modified adenine or harbor a thymine or guanine to maintain proper mitoribosomal activity.

### m^1^A, Thymine, or Guanine at 16S rRNA Position 947 May Form Stabilizing Interactions within the Mitoribosome

To interpret the structural basis of our functional observations, we modeled adenine, m^1^A, or uridine at 16S rRNA position 947 within the recently solved cryo-EM structures of the human and porcine 55S mitoribosome [17,18]. Interestingly, m^1^A947 resides within helix 71 (H71) in domain IV of the 39S large ribosomal subunit, which is located at the subunit interface near intersubunit bridge B3, where interactions with the 28S small ribosomal subunit are formed (Fig 4). The loop region of H71 interacts with H92 in 16S rRNA domain V to form an interdomain interaction (S4 Fig), which is likely stabilized by the methylation of U2552 of H92 in *E*. *coli* (human mitochondrial U1369, porcine mitochondrial U1373) [32]. Accordingly, m^1^A947 may also be involved in stabilizing the tertiary rRNA interactions in this region of the ribosome. m^1^A may do so by forming stabilizing electrostatic interactions between the positive charge induced by N^1^-methylation and the negatively charged phosphate rRNA backbone of H64 (Fig 4). The unmodified adenine, in contrast, lacks this positive charge and may not be able to provide these stabilizing interactions. These observations raise the possibility that m^1^A enrichment in mitochondrial ribosomes is due to its contribution to the formation of more stable ribosomal subunits. In the case of the *E*. *coli* ribosome, which contains a guanine at the corresponding position, the exocyclic amino group of this guanine residue may act as a hydrogen bond donor to the backbone of the 23S rRNA.

Examination of the model (and the superimposed *E*. *coli* ribosomal structure) shows that in addition to m^1^A, uridine may also form some stabilizing interactions that an unmodified adenine is unable to provide. This possibly explains why 10% of vertebrates harbor only thymine in their mtDNA and uridine in the corresponding 16S rRNA. More specifically, due to the size difference between adenine and uridine, the uridine may interact with the rRNA backbone indirectly via a water molecular bridge, reducing the effect of the partial negative charge of its exocyclic oxygen atoms. Hence, stabilization by the uridine may occur via a different mechanism from the fully-charged m^1^A modification.

## Discussion

Here we report that the non-canonical RDDs in mitochondrial 16S rRNA position 947 result from an m^1^A modification generated by the mitochondrial RNA methyltransferase TRMT61B. Thereby, our finding sets the basis for assessing the prevalence of the underlying m^1^A modification echoed by A-to-U/G RDDs throughout the genome [33]. TRMT61B introduces m^1^A modifications in position 58 of the T-loops of six tRNA species in bovine mitochondria (S5A Fig) [16,24]. Thus, similar to the bacterial RlmN methyltransferase [34], TRMT61B modifies both tRNA and rRNA and, hence, constitutes the first vertebrate methyltransferase that modifies both tRNA and rRNAs.

Alignment of the T-loops of these six tRNAs revealed a weak consensus sequence (YMRAW) surrounding m^1^A58 (S5B Fig), which is also present in the loop capping 16S rRNA H71 (UAAAU) (S5B Fig). Close inspection of the tRNAs and the 16S rRNA revealed similarity in the structure of the backbone loop and orientation of the methylated base (S6 Fig). In vitro reconstitution of m^1^A947 using recombinant TRMT61B in combination with total RNA extracted from siRNA-treated HeLa cells revealed the capability of TRMT61B to recognize deproteinized naked 16S rRNA as a substrate to introduce m^1^A947 in Helix 71. This finding indicates that m^1^A947 is likely introduced at the early assembly stage of the mitoribosomal 39S subunit. Therefore, TRMT61B likely recognizes its tRNA and rRNA targets by a similar molecular mechanism.

Notably, we observed partial reduction in the methylation state of 16S rRNA position 947 in human cells (Fig 2). Such a partial phenotype is likely due to the stability of tRNA and rRNA molecules, which have relatively long half-lives as compared to mRNA molecules. Thus, pools of tRNA and rRNA molecules are not completely replaced with newly-synthesized hypomodified ones during cultivation after knockdown, even if the knockdown efficiency is quite high. Alternatively, although TRMT61B is the first methyltransferase that introduces an m^1^A modification in both mitochondrial tRNA and 16S rRNA, we cannot exclude the possible involvement of other additional enzymes. Therefore, our results underline the importance of a future detailed analysis of the mitochondrial m^1^A methylation mechanism and possible screen for its underlying components.

Our study revealed three changes that have occurred independently at the loop capping rRNA H71, which served as convergent evolutionary solutions for the ribosomal large subunit to allow formation of fully active ribosomes: one solution is generated post-transcriptionally (the 16S rRNA 947 m^1^A modification), and the two other solutions occurred at the DNA level (thymine in 10% of the vertebrate mitoribosomes and guanine in most bacteria). Our structural analysis suggests that in contrast to the unmodified adenine, the presence of m^1^A947 or an unmodified uridine in this position of the mitoribosomal 16S rRNA or a guanine in the corresponding position of the 23S bacterial rRNA may create stabilizing interactions within the ribosome, thus likely explaining their importance for protein translation in mitochondria and bacteria. Once a reconstituted in vitro human mitochondrial translation system is available, one will be able to assess the functional impact of mitochondrial 16S rRNA mutants, wild-type, and RNA-modified molecules and study their importance for mitochondrial translation.

It is intriguing that most vertebrate mitochondrial ribosomes rely on an rRNA modification, while a nucleotide compatible with a fully active ribosome is already encoded by the bacterial gene of the large rRNA subunit, the cytosolic ribosome in eukaryotes, and in a subset of vertebrate mitochondria. Intuitively, mutations at the DNA level (the vertebrate thymine and bacterial guanine) seem like more elegant solutions, eliminating the need for RNA modification at 16S rRNA position 947. Since the adenine (which is modified at the RNA level) was retained in 90% of the vertebrates, there might be selective pressure in favor of this base at the DNA level. Three possible explanations emerge: (A) The mitochondrial 16S rRNA transcript has a second role in addition to its role in the mitoribosome, which requires the 947A. (B) Random mutagenesis led to an adenine, which, in turn, has been retained due to co-occurrence with flanking sequences that together completed the recognition motif of TRMT61B. (C) The adenine is maintained at this mtDNA position because it strengthened a yet-to-be defined regulatory element. Interestingly, recent findings may favor the third hypothesis: Recently, ChIP-seq and DNase-seq analyses enabled us to identify candidate regulatory elements even within coding mtDNA sequences, thus suggesting a dual role for such sequences [35]. Hence, the assessment of the putative regulatory impact of this region (with or without the mutations) merits further investigation.

In summary, our findings unveiled that the previously reported RDDs at 16S rRNA position 947 marked an m^1^A RNA modification introduced by the mitochondrial methyltransferase TRMT61B. As this modification is present in most 16S rRNA in the mature mammalian mitoribosome and occurs throughout vertebrate evolution, modification of position 947 is most likely important for mitoribosomal structure and function. In agreement with this idea, our bacterial model experiments indicate that in the absence of methylation, the adenine at position 947 had to be mutated (A-to-T) at the DNA level to enable translation and cell growth, as also observed in 10% of vertebrate species. As G in this position is likely the ancestral state, and is compatible with efficient protein translation, m^1^A or U likely evolved later at this position to meet the specific requirements of mitochondrial function. Finally, it is intriguing that the expression of TRMT61B is altered in Alzheimer’s disease, thus suggesting altered levels of mitochondrial tRNA and rRNA modifications in this disease [36]. In summary, three alternative evolutionary solutions (i.e., RNA modification and either of two DNA bases) were selected to maintain ribosomal function in bacteria and mitochondria.

## Materials and Methods

### Cell Culture

HeLa cells were grown in Dulbecco’s modified Eagle medium (DMEM) supplemented with 10% fetal bovine serum at 37°C, under a humidified atmosphere with 5% CO_2_.

### Purification of Mitochondrial 16S rRNA from HeLa Cells

The C-terminal Flag-tagged MRPL44 (MRPL44-Flag) expression vector was generated by LR reaction of Human Gateway Entry Clone FLJ12701AAAF with pDEST 12.2 Flag [37]. Approximately 2 × 10^8^ HeLa cells were transfected with MRPL44-Flag expression vector using FuGENE HD (Roche). At 40 h post-transfection, the cells were harvested, and 39S subunit of mitoribosome was immune-precipitated by anti-Flag M2 agarose (Sigma) as previously described [38]. Co-precipitated RNA was extracted using TRI Pure (Roche), and mitochondrial 16S rRNA was resolved further purified by denaturing PAGE.

### RNA Mass Spectrometry

Isolated mitochondrial 16S rRNA was digested by RNase T_1_ at 37°C for 60 min in an 8-μl reaction mixture containing 20 mM ammonium acetate (pH 5.3) and 10 units/μl RNase T_1_ (Epicentre). Three quarters of the digested RNA fragments were analyzed by capillary liquid chromatography coupled with nanoelectrospray ionization linear ion trap-orbitrap hybrid mass spectrometer (LTQ Orbitrap XL, Thermo Fisher Scientific) [21,39].

### Primer Extension to Detect m^1^A947 Modification

Primer extension was conducted essentially as described previously [24]. The sequences of oligonucleotides are listed in S1 Table. The 5’ ^32^P-labeled primer (0.1 pmol) was mixed with 2.25 μg of total RNA in a 5-μL solution containing 10 mM Tris-HCl (pH 8.0) and 1 mM EDTA and incubated at 80°C for 2 min, followed by cooling down to room temperature for annealing. Then, the mixture was mixed with a 4.5-μL solution containing 2 μL of 5× FS buffer (Invitrogen), 0.25 μL of 1.5 mM dATP, dTTP, dCTP, and ddGTP mix, 0.75 μL of ddH_2_O, and 1.5 μL of 25 mM MgCl_2_. Upon addition of 0.5 μL of SuperScript III (Invitrogen), the reverse transcription was carried out for 1 h at 55°C for 16S rRNA and 47°C for tRNA^Leu(UUR)^. To terminate the reaction and digest the template RNA, the mixture was added with 0.5 μL of 4M NaOH and boiled at 95°C for 5 min, then neutralized by adding 4.5 μL of 1 M Tris-HCl (pH 5.0). The cDNAs were analyzed by 20% PAGE with 7M urea. The gel was exposed to an imaging plate, and the radiolabeled bands were visualized by FLA-7000 (FujiFilm).

### RNA Interference and Real-Time RT-PCR

Knockdown of target genes using RNAi was basically preformed as described previously [24]. The siRNAs used here are listed in S2 Table. The RNAi efficiency was checked by real-time RT-qPCR with a set of primers listed in S2 Table. Total RNA (2 μg) extracted from the knockdown cells was treated with 2 U of RQ1 DNase (Promega) to remove genomic DNA in a 20 μl 1×Reaction Buffer (Promega) at 37°C for 30 min, followed by adding RQ1 DNase stop solution (Promega) and incubated at 65°C for 15 min. The DNase-treated total RNA (2 μg) was incubated at 65°C for 5 min in a 10 μL solution containing 2.5 μM oligo(dT18) primer, 60 μM random N6 primer, and 1 mM dNTPs, then cooled on ice. Subsequently, 10-μL mixture containing 2×Transcriptor RT reaction buffer (Roche), 10 U RNase inhibitor (Roche), and 5 U Transcriptor RTase (Roche) was added to the solution. The cDNAs were synthesized in the mixture by sequential incubation at 25°C for 10 min, at 55°C for 30 min, and at 85°C for 5 min. The PCR was performed in a 20 μL mixture containing a 1 μl aliquot of the cDNA solution, 0.2 μM of each PCR primers, and 1×KAPA SYBR FAST Master Mix optimized for LightCycler480(Kapa biosystems). The thermal cycling conditions included 45 cycles of 95°C for 10 s, 58°C for 20 s, and 72°C for 1 s. Amplification of cDNA was monitored by LightCycler 480 (Roche).

### Expression and Purification of the Recombinant TRMT61B Protein

His-tagged human recombinant TRMT61B was expressed in *E*. *coli* and purified by Ni-NTA chromatography as described [24]. A fraction containing the recombinant TRMT61B was dialyzed overnight against a buffer composed of 50 mM Hepes-KOH (pH 7.5), 50 mM KCl, 5 mM MgCl_2_, 10% glycerol, and 7 mM 2-mercaptoethanol. Recombinant TRMT61B was further purified by anion exchange chromatography using Mono-Q (GE healthcare) at pH 7.5 and 50–1000 mM KCl gradient. The concentration of the purified protein was determined by the Bradford protein assay (Bio-Rad) using bovine serum albumin as a standard.

### In Vitro Reconstitution of m^1^A947 Using Recombinant TRMT61B

In vitro reconstitution of m^1^A947 was carried out essentially as described previously [24].

The 114-mer RNA segment including Helix 71 (G866-U979) of human mitochondrial 16S rRNA was transcribed in vitro using T7 RNA polymerase. Template DNA with T7 class III promoter for the 114-mer RNA segment was prepared by assembling the following DNA sequences:

5’-gctaatacgactcactataggcaccgcctgcccagtgacacatgtttaacggc-3’,

5’-gtgacacatgtttaacggccgcggtaccctaaccgtgcaaaggtagcataatcac-3’,

5’-gtgcaaaggtagcataatcacttgttccttaaatagggacctgtatgaatggctccacgagggtt-3’,

and 5’-aaccctcgtggagccattc-3’. In vitro transcription by T7 RNA polymerase was performed as described [40]. The transcript was purified by denaturing PAGE and quantified by measuring the optical density at 260 nm. The reaction mix (50 μL), consisting of 25 mM Hepes-KOH (7.5), 100 mM KCl, 2.5 mM MgCl_2_, 1 mM DTT, 1 mM Ado-Met, 1.5 μg total RNA, and 1 μM His-tagged TRMT61B, was incubated for 2 h at 37°C, followed by adding phenol-chloroform isoamylalcohol (Nacalai) to terminate the reaction. Total RNA was recovered by ethanol precipitation and subjected to primer extension as described above.

### RNA-Seq and DNA-Seq Data

RNA-Seq data (publicly available from Sequence Read Archive [SRA]) from seven vertebrate species was analyzed: (*M*. *musculus* [SRR579545], *M*. *domestica* [SRR306744], *O*. *anatinus* [SRR306726], *A*. *carolinensis* [SRR579556], *G*. *gallus* [SRR579551], *X*. *tropicalis* [SRR579560], and *T*. *nigroviridis* [SRR579565]) [41,42]. Additionally, we sequenced the following samples: RNA from HeLa cells in which TRMT61B was silenced (SRR3964513) and control cells (SRR3964514), purified total RNA (SRR3963545) or DNA (SRR3963556) from isolated *S*. *scrofa* heart mitochondria, isolated mitoribosome from the same *S*. *scrofa* sample (SRR3963521), and *C*. *chamaeleon* RNA extracted from whole blood (SRR2962875).

### Isolation of Mitochondria from Porcine Liver Tissue

Porcine mitochondria were prepared from a liver sample extracted from a single freshly slaughtered pig (*S*. *scrofa*). The preparation was done as previously described by Greber et al. [43]. To avoid contamination from other liver samples, only one liver was processed at a time. The volumes of buffers used at different steps during the preparation were reduced according to the protocol that indicates the volumes for a preparation of five livers.

### Preparation of 55S Mitoribosomes

The preparation of the 55S mitoribosome follows the procedure previously described by Greber et al. [43]. Sixty-six grams of frozen mitoplasts were thawed in 150 ml lysis buffer (20 mM HEPES-KOH, pH 7.6, 100 mM KCl, 20 mM MgCl_2_, 1 mM dithiothreitol [DTT], 125 μM spermine, 125 μM spermidine) and brought to a total volume of 225 ml with monosome buffer (20 mM HEPES-KOH, pH 7.6, 100 mM KCl, 20 mM MgCl_2_, 1 mM DTT). Twenty-five milliliters Triton X-100 buffer (monosome buffer with 16% [v/v] Triton X-100) were added and the solution was stirred for 15 min at 4°C before homogenization using a Dounce homogenizer. The suspension was centrifuged (SLA-1500, 13,000 rpm, 20 min, 4°C), and the supernatant was PEG precipitated in 5% (w/v) PEG 10,000 for 15 min. The precipitate was collected by centrifugation (Sorvall SLA-3000 [Thermo Fisher Scientific], 2,500 g, 7 min, 4°C). Each pellet was re-suspended in 35 ml monosome buffer (2 h shaking), and the suspension was homogenized using a Dounce homogenizer before centrifugation using a Beckman (Beckman-Coulter) Type 45Ti rotor (28,000 rpm, 17 min, 4°C). The supernatant was loaded onto 50% (w/v) sucrose cushions (15 ml) and centrifuged (Beckman Type 70Ti [Beckman-Coulter], 50,000 rpm, 24 h, 4°C). Pellets were dissolved in 500 μl monosome buffer (shaking 230 rpm, 1 h) and cleared (tabletop centrifuge, 16,000 rpm, 20 min, 4°C). The sample was distributed onto 10%–40% (w/v) sucrose gradients (1 ml per gradient) and centrifuged (Beckmann SW-32 Ti [Beckman-Coulter], 26,000 rpm, 12 h, 4°C). The gradients were fractionated, and fractions corresponding to the 55S mitoribosome were collected and pooled. 55S mitoribosomes were pelleted using a Beckman (Beckman-Coulter) Type TLA-55 rotor (50,000 rpm, 6 h, 4°C). The supernatant was immediately discarded, and the pellets were flash-frozen in liquid nitrogen.

### DNA and RNA Extraction from *S*. *scrofa* and *C*. *chamaeleon* Samples

DNA was extracted using the Genomics DNA Extraction Mini Kit (RBC Bioscience), and RNA was extracted using the Perfect Pure RNA Cell and Tissue Kit (5 PRIME), following the manufacturers’ protocol. RNA and DNA were extracted from isolated mitoplasts, and RNA was purified from 55S mitoribosomes and blood of a single *C*. *chamaeleon*. The *C*. *chamaeleon* sample was collected as part of a different study in our lab [44]. The sample was collected and returned to its capturing site (UTM coordinates: 681839.21E/3597036.47N) using permits from the Israel Nature and Parks authority, number 2013/40003, and was approved by the animal experiments board at Ben-Gurion University number IL-18-03-2012.

### cDNA Synthesis

One microgram of total RNA was subjected to cDNA synthesis using the iScript cDNA Synthesis Kit (Bio-Rad), following the manufacturer’s protocol.

### Massive Parallel Deep Sequencing

DNA libraries were prepared using the Nextera XT DNA Sample Preparation Kit (Illumina). RNA libraries were prepared using the TruSeq RNA Kit (Illumina) according to the manufacturer’s protocol. TRMT61B-si and control-si RNA samples were sequenced using the Illumina HiSeq 2500 platform (Technion Genome Center, Israel) with 50-nt single-end reads. Both *S*. *scrofa* DNA and RNA libraries were sequenced using the MiSeq platform (Illumina). DNA and RNA libraries were sequenced using 151-nt or 300-nt, paired-end reads. *C*. *chamaeleon* libraries were sequenced on Hi-Seq 2000 platform (Illumina) using 101-nt paired end reads.

### Analysis of Massive Parallel Sequencing Data

Sequencing reads were aligned against the publicly available mtDNA sequence of each species (*M*. *musculus*: NC_005089.1, *M*. *domestica*: NC_006299.1, *O*. *anatinus*: NC_000891.1, *A*. *carolinensis*: NC_010972.2, *G*. *gallus*: NC_007236.1, *X*. *tropicalis*: NC_006839.1, *T*. *nigroviridis*: NC_007176.1, *S*. *scrofa*: NC_000845.1, and *C*. *chamaeleon*: JF317641.1). For multiple sequence alignment, we utilized BWA [45] following the default protocol of the 1,000 Genome Sequence Analysis (ftp.1000genomes.ebi.ac.uk/vol1/ftp/). Only reads that were aligned to the corresponding mtDNA were used for further analyses. SAMtools [46] was used to convert the SAM to the BAM sequence format. MitoBam Annotator [47] was used to identify secondary read changes in the corresponding RNA sample. The orthologous sequences of the human mtDNA sequence position 2617 in each of the tested species were identified and analyzed. Secondary read changes were considered high quality only if identified in at least 1,000 high-quality sequence reads (filter A, except *M*. *domestica*), if their minimal read fraction was at least 1.6% (i.e., 0.8% from the reads of each of the strands [filter B] [48], and after manual inspection using the Integrative Genomics Viewer [49] to exclude mutations at the edges of the reads (S7 Fig).

### Multiplex Automated Genome Engineering (MAGE)

In order to mutate the 947 orthologous position in all seven rRNA (23S) genes (position 1954), we used the *E*. *coli* strain EcM2.1 (a specially designed strain for high MAGE efficiency) to carry out three successive MAGE cycles as previously described [31]. We used two 90bp single-strand oligonucleotides to target the lagging strand of all seven genes. The first oligo was used to replace the endogenous G with an A: G*T*GGAGACAGCCTGGCCATCATTACGCCATTCGTGCAGGTCGGAA**T**TTACCCGACAAGGAATTTCGCTACCTTAGGACCGTTATAGTT*A*C, and the second to replace the endogenous G with a T: G*T*GGAGACAGCCTGGCCATCATTACGCCATTCGTGCAGGTCGGAA**A**TTACCCGACAAGGAATTTCGCTACCTTAGGACCGTTATAGTT*A*C. The mutated base is underlined. Asterisks represent phosphorothioate bonds. Briefly, cells were grown overnight at 34°C. Then, 30 μl of the saturated culture was transferred into fresh 3 ml of LBL medium until reaching OD = 0.4 and then moved to a shaking water bath (350 RPM) at 42°C for 15 min, after which it was moved immediately to ice. Next, 1 ml was transferred to an Eppendorf tube, and cells were washed twice with ddW at centrifuge speed of 13,000 g for 30 s. Next, the bacterial pellet was dissolved in 50 μl of DDW containing 2 μM of SS-DNA oligo and transferred into a cuvette. Electroporation was performed in 1.78 kV, 200 ohms, 25 μF. After electroporation, the bacteria were transferred into 2 ml of fresh LBL and incubated in 34°C until again reaching OD = 0.4 for an additional MAGE cycle.

### Identification of Positive MAGE Colonies by RFLP Analyses and Sanger Sequencing

To identify positive MAGE colonies (referred to as bacterial strains throughout the text), we PCR amplified two fragments encompassing the bacterial genomic regions (*E*. *coli*) orthologous to position 2,617 in all seven large rRNA (23S) genes. The amplified fragments correspond to *E*. *coli* genome (gi|556503834:4168641–4171544 [*rrlB*] positions 1,929–2,043 [fragment one] and to positions 1,929–2,333 [fragment two]). Restriction fragment length polymorphism (RFLP) was conducted on fragment one using MlucI (New England Biolabs—#R0538S) to identify the G-to-A or G-to-T mutations (both changes created a MlucI restriction site). Thus, complete restriction digestion of fragment one products implied genome editing (i.e., from wild type G to either T or A) in all seven copies of the 23S gene. To verify this interpretation, we amplified fragment two in samples showing complete digestion of fragment one. We then purified and sequenced those samples using primer 3 (S3 Table 3 and S4 Table). These sequences were aligned and visualized using Sequencher 4.10 (GeneCodes). Furthermore, after the initial screen, we PCR amplified each of the seven 23S rRNA genes by a set of specific primer combinations (S3 and S4 Tables) to ensure that the resulting modified strains harbor the desired mutation in all seven genes. To this end, the gene-specific templates were created by 50X dilution of the original PCR product of fragment 1, from which 1 μl was used as template for a new PCR amplification using primer pairs specific to each of the seven 23S genes. The resulting gene-specific amplification products were subjected to restriction digestion by MlucI, as mentioned above. All primers, PCR, and RFLP reactions and conditions are described in S3 Table 3 and S4 Tables. PCR and RFLP products were visualized by an EtBr-stained 1% agarose gel. PCR fragments were purified using Wizard SV Gel and PCR Clean-up system (Promega), following manufacturer’s protocol, and sequenced at the BGU sequencing core facility.

### Liquid Growth Measurements

Cultures were grown for 48 h in LB medium, back diluted in a 1:100, ratio and dispensed on 96-well plates. Wells were measured for optical density at OD_600_, and measurements were taken during the growth at 30min intervals until reaching stationary phase. Qualitative growth comparisons were performed using 96-well plates (Thermo Scientific). For each strain, a growth curve was obtained by averaging over 48 wells.

### In Vivo Translation Examination

Strains were transformed with the plasmid pZS*11-YFP-CGC-Kan harboring a YFP gene and Kan resistance cassette. YFP was measured as described in the section “Liquid Growth Measurements.” YFP production rate was measured by subtracting the YFP value at time t by time t-1 and dividing this value by the OD value at time t. Maximal production rate was defined as the highest value of this graph, and total production is the area under it.

### In Vitro Translation Examination

Each of the MAGE-treated *E*.*coli* strains has been subjected to a 30S cell extract protocol [50]. All strains were grown to O.D_600_ 2.0 ± 0.05, then lysed according to the 30S cell extract protocol while carefully maintaining all the strains under the same exact conditions throughout all processes. Next, cell extracts were used for a cell-free protein synthesis assay using EGFP fluorescence as a reporter. The assay was conducted in a Nunc 384 (120 μL) well plates (Thermo Fisher Scientific, Waltham, MA) and was monitored using time-dependent florescence measurements using a plate reader (Excitation 485 nm, Emission 525 nm). A typical cell-free reaction assay consists of 10 μL reaction mixture containing 33% (by volume) *E*. *coli* cell extract, and 66% of the reaction volume is composed of the reaction buffer containing nutrients, metabolites, and crowding agents. The reporter plasmid (pBEST-OR2-OR1-Pr-UTR1-deGFP-T500 [Addgene #40019]) is finally added to final concentration of 2nM. For detailed methodology, please see [51].

## Supporting Information

S1 Data Exact values for data presented in Figs 2C, 3A and 3B, 5A, 5B and 5C. (XLSX)

S1 Fig Mass spectrometric analysis of RNase T_1_-digested fragments of mitochondrial 16S rRNA to detect methylated residues. Three previously identified methylations in 16S rRNA were confirmed in this analysis. The first panel shows a base peak chromatogram (BPC). The second panel represents mass chromatogram for detecting triply-charged negative ion of the di-methylated 11-mer fragment containing Um1369 and Gm1370 (UUUmGmUUCAACGp, m/z 1177.2). The third panel represents mass chromatogram for detecting singly-charged negative ion of dimer containing Gm1145 (GmGp, m/z 721.1). Triangles represent authentic fragments confirmed by checking their mass spectra.
(TIF)

S2 Fig CID spectrum of the methylated tetramer in the 114-mer RNA segment. RNase A-digested fragments of the methylated 114-mer RNA segment was analyzed by capillary LC/nano-ESI-MS. The precursor ion for CID was *m/z* 1324.20. The sequence was confirmed by assignment of the product ions. Nomenclature for the product ions is in accordance with a previous report [22].
(TIF)

S3 Fig Using MAGE to mutate position 1954 in *E*. *coli* 23S genes. Sanger sequencing of representative strains was employed to identify mutants from G (WT) into either T or A. Red arrow points at the orthologous position 947 of the 16S rRNA (position 1954) in *E*. *coli*.
(TIF)

S4 Fig Interdomain interaction (dotted lines) between H71 and H92 in *E*. *coli* 23S rRNA (A) and in human mitochondrial 16S rRNA (B). Bases involved in bridge B3 are colored red. Post-transcriptional modifications are shown in blue.
(TIF)

S5 Fig (A) Secondary structure of T-loop in bovine mitochondrial tRNA species bearing m^1^A58. m^1^A58 is colored red. (B) Alignment of T-loop sequences from bovine mitochondrial tRNAs and H71 loop sequence in human mitochondrial 16S rRNA. Position 58 is highlighted in red.
(TIF)

S6 Fig Overlap of tRNA stem-and-loop on ribosomal H71. Sticks-and-ribbon representation of structural overlap between helices H71 in *S*. *scrofa* (brown) porcine mitoribosomal large subunit (PDB accession code 4v1a and 4v19) and tRNA-Phe (purple, PDB accession code 3TUP). In sticks the overlapping position of A58 and A947, which are the target of the methylation by TRMT61B.
(TIF)

S7 Fig The identified RDDs are not found at the edges of the sequence reads. IGV viewer chart [47] at orthologous position 947 of human 16S rRNA in the analyzed samples harboring the RDDs. Upper panel: schematic linear representation of the mtDNA in each of the tested species. Framed: position 947. Numbers at the top: nucleotide positions of each species mtDNA. Lower large panel: schematic representation of the sequence reads encompassing 16S orthologous position 947 (thick arrow-like grey bars). Direction of arrow heads correspond to sequencing read directions. Bar colors: red: thymine, brown: guanine, grey: adenine. A: *A*. *carolinensis*, B: *C*. *chamaeleon*, C: *G*. *gallus*, D: *M*. *domestica*, E: *T*. *nigroviridis*, F: *X*. *tropicalis*, G: *S*. *scrofa* (RNA from pure mitochondrial sample), and H: *S*. *scrofa* (RNA from pure mitoribosome sample).
(TIF)

S1 Table List of siRNAs used in this study. (DOCX)

S2 Table List of DNA primers used in RT-qPCR and primer extension. (DOCX)

S3 Table PCR amplification and sequencing primers. (DOCX)

S4 Table PCR and RFLP reactions mix and conditions. (DOCX)

## Abbreviations

GFP: green fluorescent protein
m^1^A: 1-methyladenosine
mtDNA: mitochondrial DNA
RDD: RNA-DNA difference
YFP: yellow fluorescent protein
