# Correction: Mitochondrial 16S rRNA Is Methylated by tRNA Methyltransferase TRMT61B in All Vertebrates

**DOI:** 10.1371/journal.pbio.1002594

**Published:** 2017-01-19

**Authors:** Dan Bar-Yaacov, Idan Frumkin, Yuka Yashiro, Takeshi Chujo, Yuma Ishigami, Yonatan Chemla, Amit Blumberg, Orr Schlesinger, Philipp Bieri, Basil Greber, Nenad Ban, Raz Zarivach, Lital Alfonta, Yitzhak Pilpel, Tsutomu Suzuki, Dan Mishmar

## Notice of Republication

This article was republished on December 30, 2016, to correct errors in the reference list and corresponding citations, which were unintentionally included from an earlier draft. Please download this article again to view the correct version. The originally published, uncorrected article and the republished, corrected articles are provided here for reference.

In addition, the superscript formatting in “AAm^1^AUp” and “tRNALeu^(UUR)^”, the subscript formatting in “MgCl_2_”, and a typographical error in “methyltransferase” have been corrected throughout the manuscript. There was also an error in the sentence describing Fig 2A; the correct sentence is: “In contrast, knock down of TRMT10C did not lead to altered cDNA extension”.

The following references have been replaced:

**10.** Larson ET, Kim JE, Zucker FH, Kelley A, Mueller N, Napuli AJ, et al. Structure of Leishmania major methionyl-tRNA synthetase in complex with intermediate products methionyladenylate and pyrophosphate. Biochimie. 2011; 93(3):570–82. doi: 10.1016/j.biochi.2010.11.015 PMID: 21144880 is now **10.** Piskol R, Peng Z, Wang J, Li JB. Lack of evidence for existence of noncanonical RNA editing. Nature biotechnology. 2013;31(1):19–20.

**41.** Gershoni M, Levin L, Ovadia O, Toiw Y, Shani N, Dadon S, et al. Disrupting Mitochondrial—Nuclear Coevolution Affects OXPHOS Complex I Integrity and Impacts Human Health. Genome Biology and Evolution. 2014; 6(10):2665–80. doi: 10.1093/gbe/evu208 PMID: 25245408 is now **41.** Brawand D, Soumillon M, Necsulea A, Julien P, Csárdi G, Harrigan P, et al. The evolution of gene expression levels in mammalian organs. Nature. 2011;478(7369):343–8.

**42.** Brown A, Amunts A, Bai X-c, Sugimoto Y, Edwards PC, Murshudov G, et al. Structure of the large ribosomal subunit from human mitochondria. Science. 2014; 346(6210):718–22. doi: 10.1126/science. 1258026 PMID: 25278503 is now **42.** Barbosa-Morais NL, Irimia M, Pan Q, Xiong HY, Gueroussov S, Lee LJ, et al. The evolutionary landscape of alternative splicing in vertebrate species. Science. 2012;338(6114):1587–93.

The following references have now been removed:

**29.** Nakanishi K, Ogiso Y, Nakama T, Fukai S, Nureki O. Structural basis for anticodon recognition by methionyl-tRNA synthetase. Nature structural & molecular biology. 2005; 12(10):931–2.

**31.** Emsley P, Cowtan K. Coot: model-building tools for molecular graphics. Acta Crystallographica Section D: Biological Crystallography. 2004; 60(12):2126–32.

**47.** (This was a duplicate of **42.**) Brown A, Amunts A, Bai X-c, Sugimoto Y, Edwards PC, Murshudov G, et al. Structure of the large ribosomal subunit from human mitochondria. Science. 2014:1258026.

The following references have now been added:

**28.** Greber BJ, Boehringer D, Leibundgut M, Bieri P, Leitner A, Schmitz N, et al. The complete structure of the large subunit of the mammalian mitochondrial ribosome. Nature. 2014;515(7526):283–6.

**30.** Noeske J, Wasserman MR, Terry DS, Altman RB, Blanchard SC, Cate JH. High-resolution structure of the Escherichia coli ribosome. Nature structural & molecular biology. 2015;22(4):336–41.

**43.** Greber BJ, Boehringer D, Leitner A, Bieri P, Voigts-Hoffmann F, Erzberger JP, et al. Architecture of the large subunit of the mammalian mitochondrial ribosome. Nature. 2014;505(7484):515–9.

**44.** Bar-Yaacov D, Hadjivasiliou Z, Levin L, Barshad G, Zarivach R, Bouskila A, et al. Mitochondrial Involvement in Vertebrate Speciation? The Case of Mito-nuclear Genetic Divergence in Chameleons. Genome Biol Evol. 2015;7(12):3322–36. doi: 10.1093/gbe/evv226. PubMed PMID: 26590214; PubMed Central PMCID: PMCPMC4700957.

**49.** Robinson JT, Thorvaldsdóttir H, Winckler W, Guttman M, Lander ES, Getz G, et al. Integrative genomics viewer. Nature biotechnology. 2011;29(1):24–6.

The citation numbers of the following references have changed:

**8.** is now **9.** Wang IX, Core LJ, Kwak H, Brady L, Bruzel A, McDaniel L, et al. RNA-DNA differences are generated in human cells within seconds after RNA exits polymerase II. Cell Rep. 2014; 6(5):906–15. doi: 10.1016/j.celrep.2014.01.037 PMID: 24561252

**9.** is now **8.** Bar-Yaacov D, Avital G, Levin L, Richards AL, Hachen N, Jaramillo BR, et al. RNA—DNA differences in human mitochondria restore ancestral form of 16S ribosomal RNA. Genome research. 2013; 23 (11):1789–96. doi: 10.1101/gr.161265.113 PMID: 23913925

**17.** is now **18.** Greber BJ, Bieri P, Leibundgut M, Leitner A, Aebersold R, Boehringer D, et al. The complete structure of the 55S mammalian mitochondrial ribosome. Science. 2015; 348(6232):303–8 doi: 10.1126/science.aaa3872 PMID: 25837512

**18.** is now **17.** Amunts A, Brown A, Toots J, Scheres SH, Ramakrishnan V. The structure of the human mitochondrial ribosome. Science. 2015; 348(6230):95–8 doi: 10.1126/science.aaa1193 PMID: 25838379

**28.** is now **29.** Voorhees RM, Fernández IS, Scheres SH, Hegde RS. Structure of the mammalian ribosome-Sec61 complex to 3.4 Å resolution. Cell. 2014; 157(7):1632–43. doi: 10.1016/j.cell.2014.05.024 PMID: 24930395

**30.** is now **31.** Wang HH, Isaacs FJ, Carr PA, Sun ZZ, Xu G, Forest CR, et al. Programming cells by multiplex genome engineering and accelerated evolution. Nature. 2009; 460(7257):894–8. doi: 10.1038/nature08187 PMID: 19633652

**43.** is now **45.** Li H, Durbin R. Fast and accurate short read alignment with Burrows-Wheeler transform. Bioinformatics. 2009; 25(14):1754–60. PMID: 19451168. doi: 10.1093/bioinformatics/btp324

**44.** is now **46.** Li H, Handsaker B, Wysoker A, Fennell T, Ruan J, Homer N, et al. The Sequence Alignment/Map format and SAMtools. Bioinformatics. 2009; 25(16):2078–9. PMID: 19505943. doi: 10.1093/bioinformatics/btp352

**45.** is now **47.** Zhidkov I, Nagar T, Mishmar D, Rubin E. MitoBamAnnotator: A web-based tool for detecting and annotating heteroplasmy in human mitochondrial DNA sequences. Mitochondrion. 2011; 11(6):924–8. PMID: 21875693. doi: 10.1016/j.mito.2011.08.005

**46.** is now **48.** He Y, Wu J, Dressman DC, Iacobuzio-Donahue C, Markowitz SD, Velculescu VE, et al. Heteroplasmic mitochondrial DNA mutations in normal and tumour cells. Nature. 2010; 464(7288):610–4. PMID: 20200521. doi: 10.1038/nature08802

**48.** is now **50.** Sun ZZ, Hayes CA, Shin J, Caschera F, Murray RM, Noireaux V. Protocols for implementing an Escherichia coli based TX-TL cell-free expression system for synthetic biology. J Vis Exp. 2013;(79):e50762. doi: 10.3791/50762 PMID: 24084388; PubMed Central PMCID: PMC3960857.

**49.** is now **51.** Chemla Y, Ozer E, Schlesinger O, Noireaux V, Alfonta L. (2015). Genetically expanded cell-free protein synthesis using endogenous pyrrolysyl orthogonal translation system. Biotechnol Bioeng. 2015 Aug; 112(8):1663–72. doi: 10.1002/bit.25587 Epub 2015 Jun 16.

## Supporting Information

S1 File Originally published, uncorrected article. (PDF)

S2 File Republished, corrected article. (PDF)
