# Supplementary figures and images for: Mitochondrial 16S rRNA Is Methylated by tRNA Methyltransferase TRMT61B in All Vertebrates
Source: PLoS Biol. 2016 Sep 15;14(9):e1002557. doi: 10.1371/journal.pbio.1002557 (PMC5025228; doi:10.1371/journal.pbio.1002557)

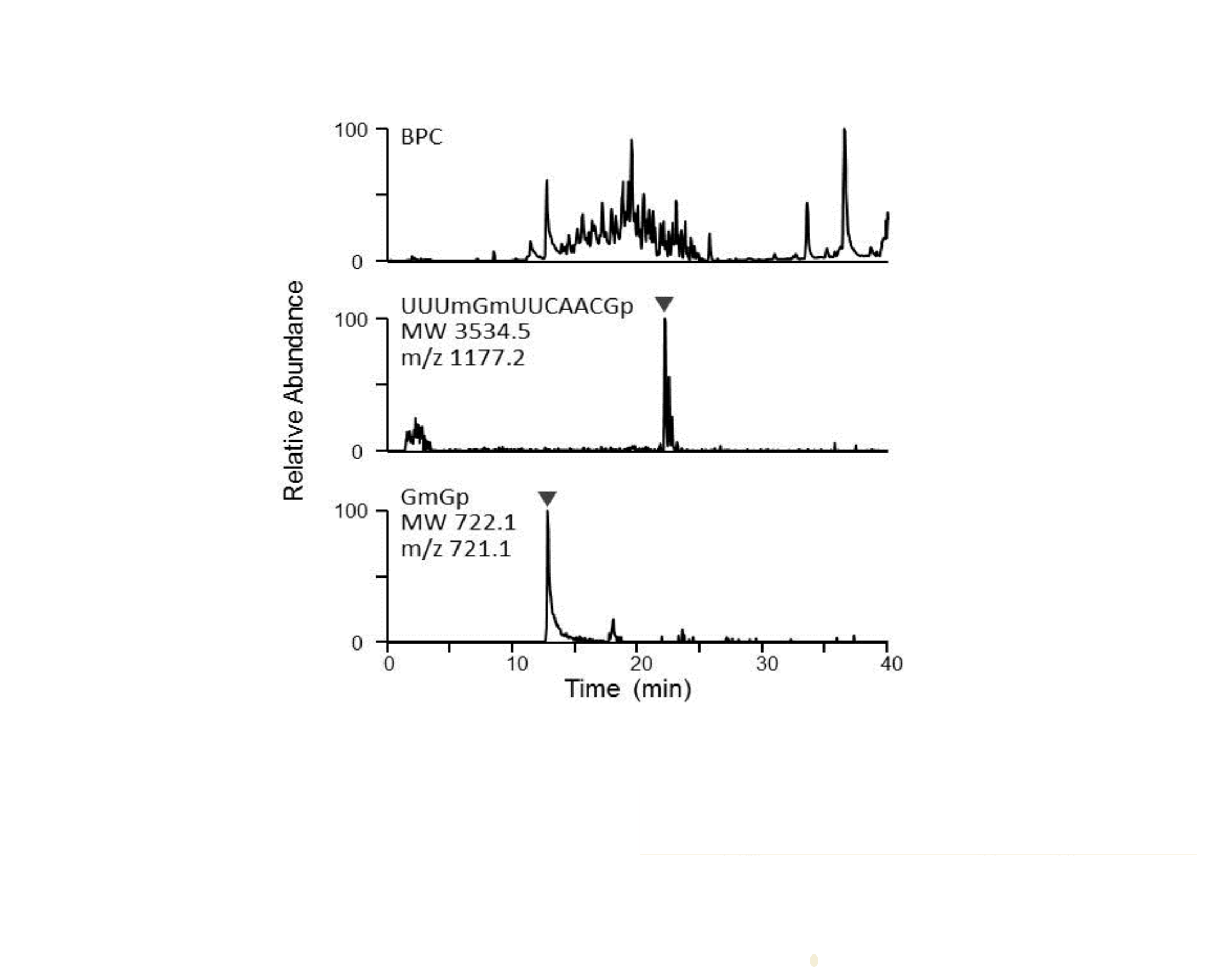

Supplement: S1 Fig — Three previously identified methylations in 16S rRNA were confirmed in this analysis. The first panel shows a base peak chromatogram (BPC). The second panel represents mass chromatogram for detecting triply-charged negative ion of the di-methylated 11-mer fragment containing Um1369 and Gm1370 (UUUmGmUUCAACGp, m/z 1177.2). The third panel represents mass chromatogram for detecting singly-charged negative ion of dimer containing Gm1145 (GmGp, m/z 721.1). Triangles represent authentic fragments confirmed by checking their mass spectra. (TIF) [file pbio.1002557.s002.tif]

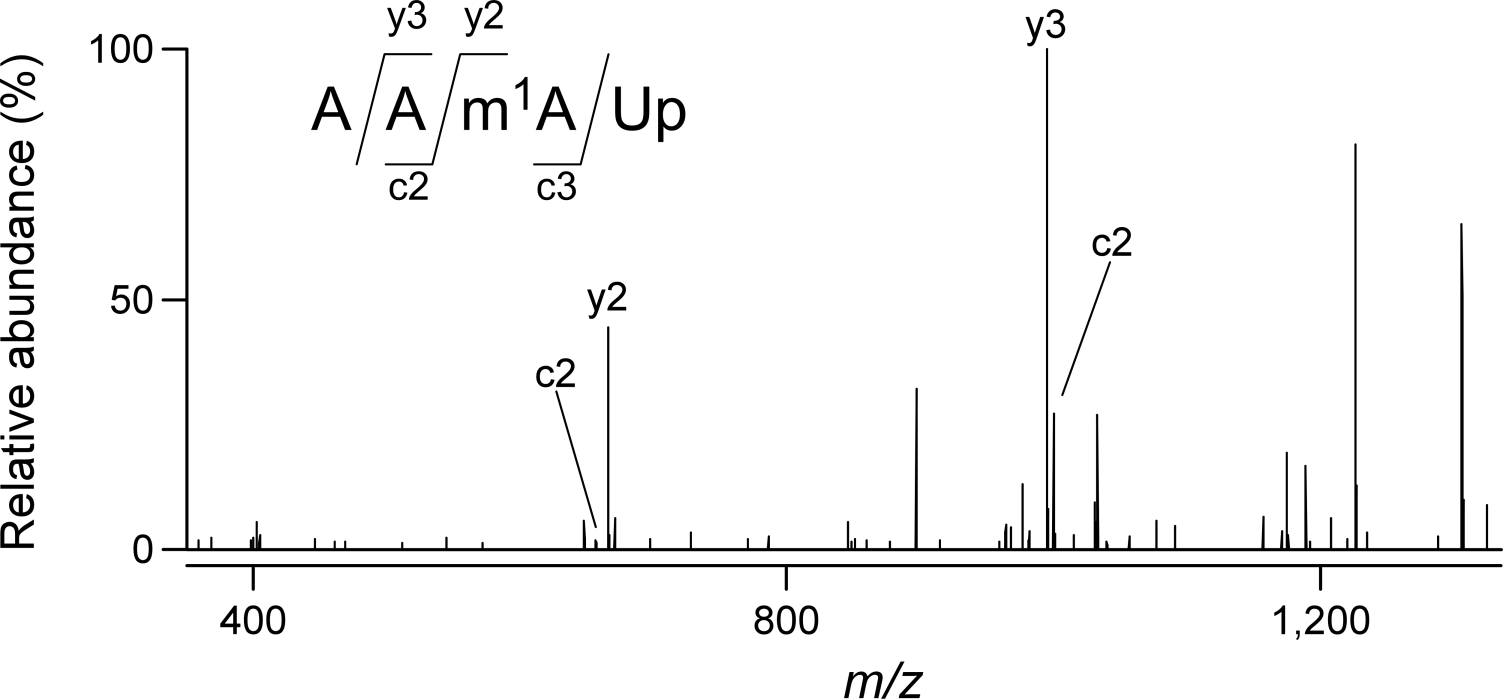

Supplement: S2 Fig — RNase A-digested fragments of the methylated 114-mer RNA segment was analyzed by capillary LC/nano-ESI-MS. The precursor ion for CID was m/z 1324.20. The sequence was confirmed by assignment of the product ions. Nomenclature for the product ions is in accordance with a previous report [22]. (TIF) [file pbio.1002557.s003.tif]

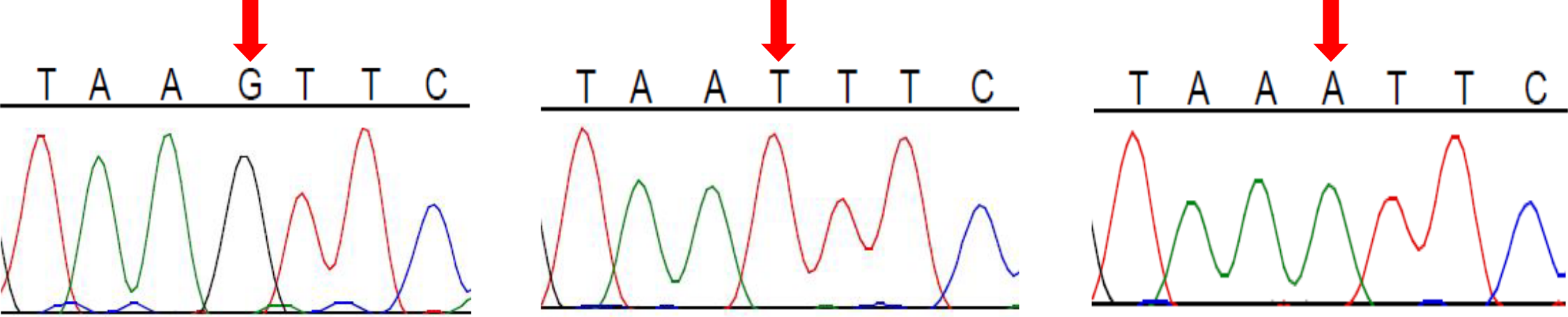

Supplement: S3 Fig — Sanger sequencing of representative strains was employed to identify mutants from G (WT) into either T or A. Red arrow points at the orthologous position 947 of the 16S rRNA (position 1954) in E. coli. (TIF) [file pbio.1002557.s004.tif]

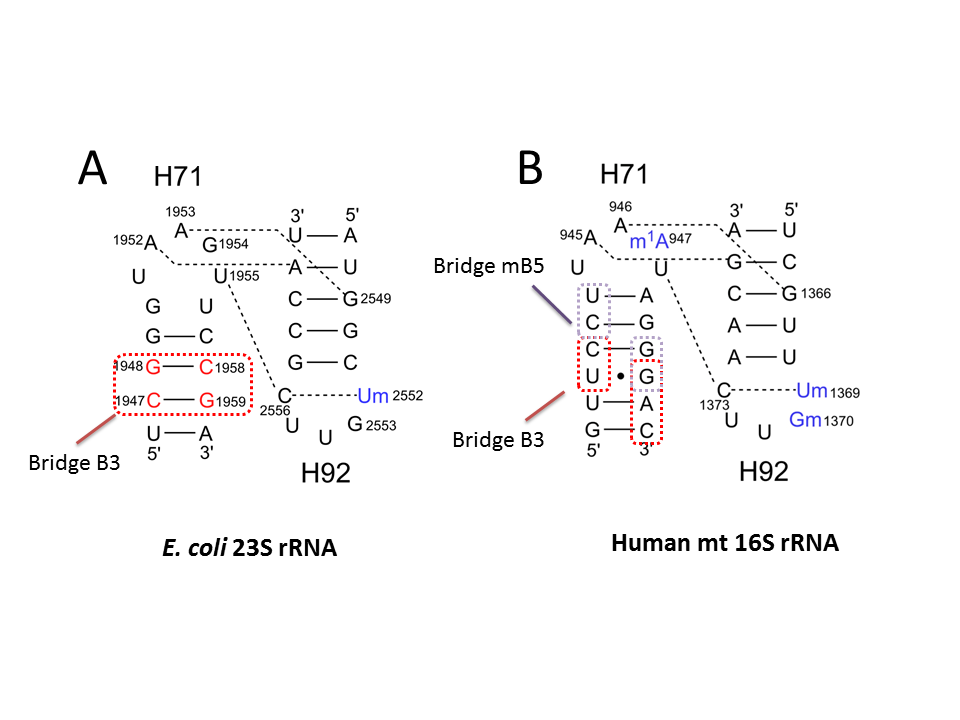

Supplement: S4 Fig — Bases involved in bridge B3 are colored red. Post-transcriptional modifications are shown in blue. (TIF) [file pbio.1002557.s005.tif]

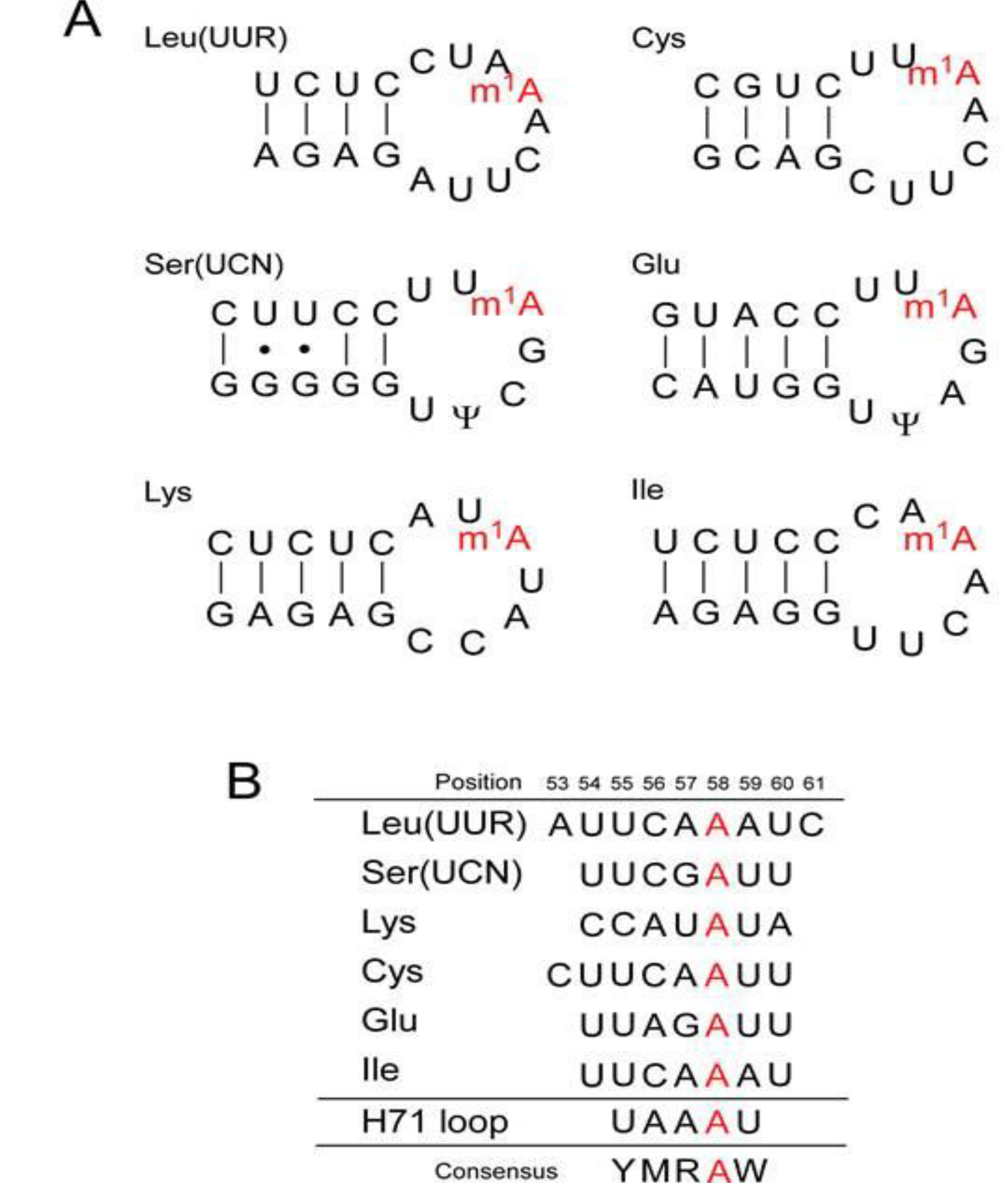

Supplement: S5 Fig — (A) Secondary structure of T-loop in bovine mitochondrial tRNA species bearing m1A58. m1A58 is colored red. (B) Alignment of T-loop sequences from bovine mitochondrial tRNAs and H71 loop sequence in human mitochondrial 16S rRNA. Position 58 is highlighted in red. (TIF) [file pbio.1002557.s006.tif]

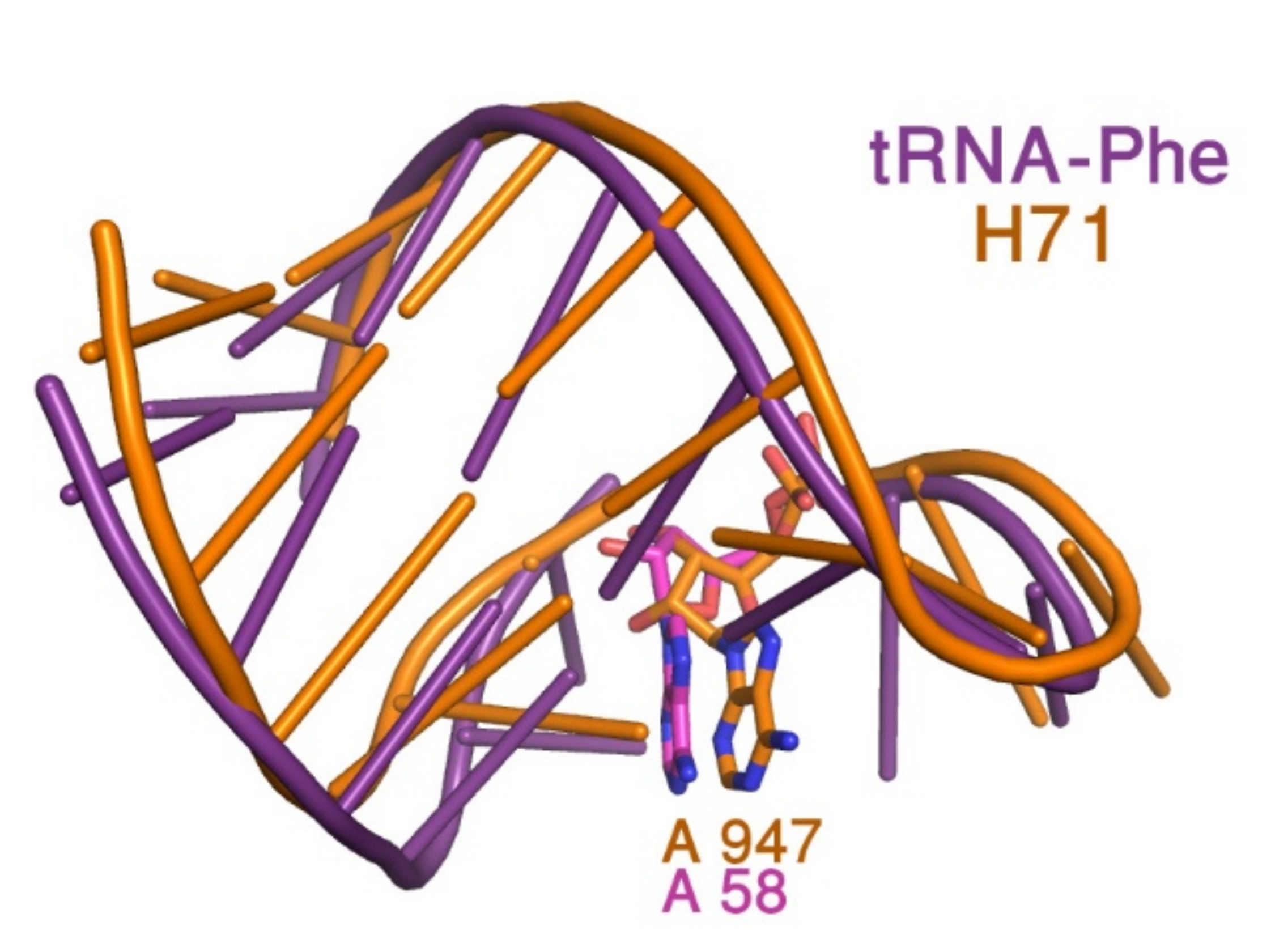

Supplement: S6 Fig — Sticks-and-ribbon representation of structural overlap between helices H71 in S. scrofa (brown) porcine mitoribosomal large subunit (PDB accession code 4v1a and 4v19) and tRNA-Phe (purple, PDB accession code 3TUP). In sticks the overlapping position of A58 and A947, which are the target of the methylation by TRMT61B. (TIF) [file pbio.1002557.s007.tif]

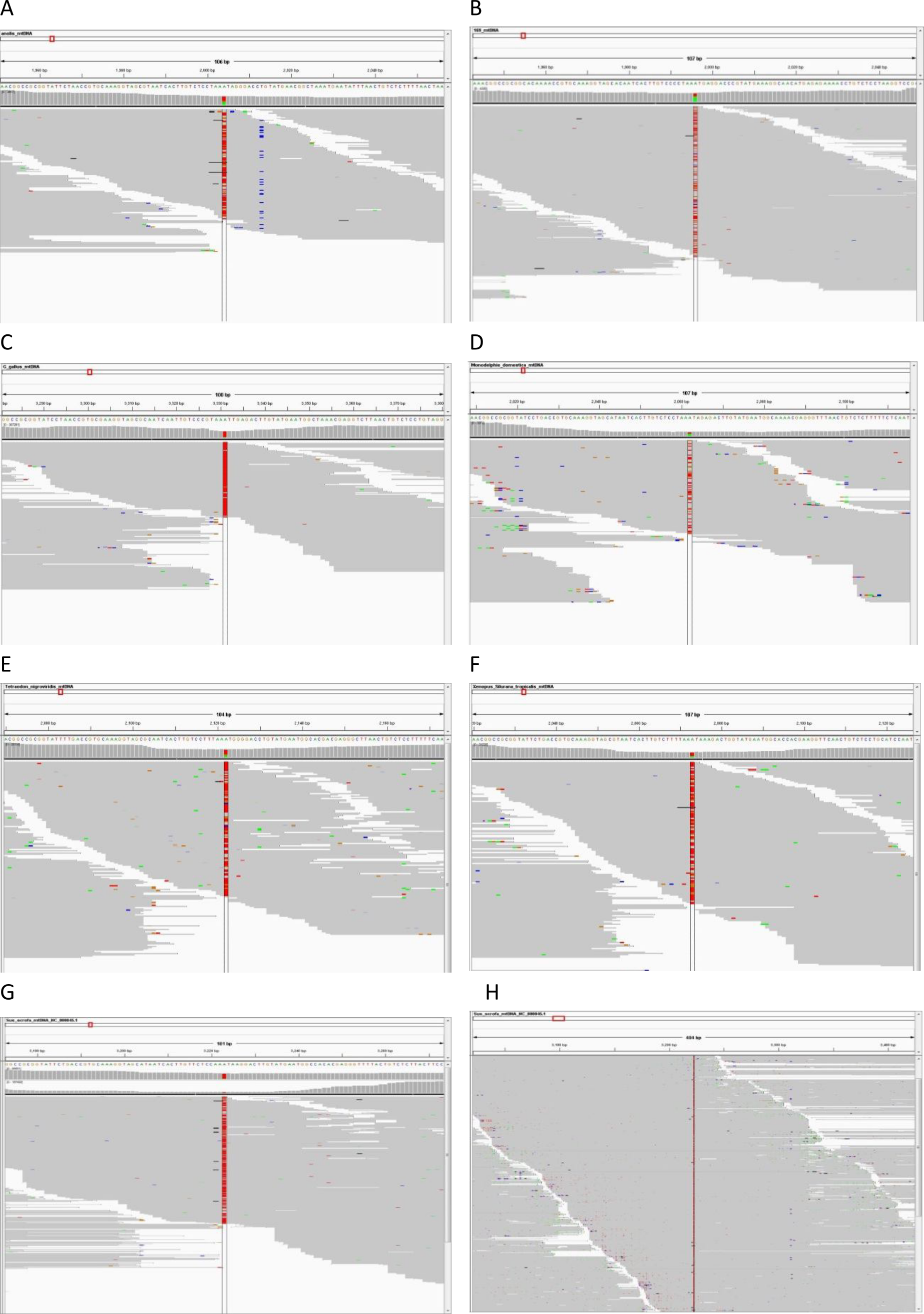

Supplement: S7 Fig — IGV viewer chart [47] at orthologous position 947 of human 16S rRNA in the analyzed samples harboring the RDDs. Upper panel: schematic linear representation of the mtDNA in each of the tested species. Framed: position 947. Numbers at the top: nucleotide positions of each species mtDNA. Lower large panel: schematic representation of the sequence reads encompassing 16S orthologous position 947 (thick arrow-like grey bars). Direction of arrow heads correspond to sequencing read directions. Bar colors: red: thymine, brown: guanine, grey: adenine. A: A. carolinensis, B: C. chamaeleon, C: G. gallus, D: M. domestica, E: T. nigroviridis, F: X. tropicalis, G: S. scrofa (RNA from pure mitochondrial sample), and H: S. scrofa (RNA from pure mitoribosome sample). (TIF) [file pbio.1002557.s008.tif]
