# Supplementary material for: Mitochondrial 16S rRNA Is Methylated by tRNA Methyltransferase TRMT61B in All Vertebrates
Source: PLoS Biol. 2016 Sep 15;14(9):e1002557. doi: 10.1371/journal.pbio.1002557 (PMC5025228; doi:10.1371/journal.pbio.1002557)
Supplement: S1 Table — (DOCX) [file pbio.1002557.s009.docx]

**Supplementary Table 1**

**List of siRNAs used in this study.**

| siRNA | Sequense |
| --- | --- |
| Luciferase sense | cguacgcggaauacuucgaag |
| Luciferase antisense | ucgaaguauuccgcguacgau |
| TRMT61B si sense | ggagcaaccgaagacauaaag |
| TRMT61B si antisense | uuaugucuucgguugcuccau |
| TRMT10C si sense | guuuccaggaucaaaacuaag |
| TRMT10C si antisense | uaguuuugauccuggaaacau |
