# Supplementary material for: Mitochondrial 16S rRNA Is Methylated by tRNA Methyltransferase TRMT61B in All Vertebrates
Source: PLoS Biol. 2016 Sep 15;14(9):e1002557. doi: 10.1371/journal.pbio.1002557 (PMC5025228; doi:10.1371/journal.pbio.1002557)
Supplement: S2 Table — (DOCX) [file pbio.1002557.s010.docx]

**Supplementary Table 2**

**List of DNA primers used in RT-qPCR and primer extension**

| oligo DNA | Sequence |
| --- | --- |
| ACTB Fw | ctggcaccacaccttctac |
| ACTB Rv | ggcatacccctcgtagatg |
| TRMT61B Fw | atttcaggagcaaccgaaga |
| TRMT61B Rev | caaggcaaaccaaccaatct |
| TRMT10C Fw | ccttctgtatgccacactgc |
| TRMT10C Rv | gtctccagttgtttgcacca |
| mt 16S rRNA_954 | cctcgtggagccattcatacagg |
| mt tRNA Leu (UUR) | tggtgttaagaagagg |
