# Supplementary material for: Mitochondrial 16S rRNA Is Methylated by tRNA Methyltransferase TRMT61B in All Vertebrates
Source: PLoS Biol. 2016 Sep 15;14(9):e1002557. doi: 10.1371/journal.pbio.1002557 (PMC5025228; doi:10.1371/journal.pbio.1002557)
Supplement: S3 Table — (DOCX) [file pbio.1002557.s011.docx]

**Supplementary Table 3**

| **Number** | **Species** | **Start position** | **Sequence** |
| --- | --- | --- | --- |
| 1 | *Escherichia coli* | *rrlB* position - 1929 | GGTAGCGAAATTCCTTGTCGGG |
| 2 | *Escherichia coli* | *rrlB* position - 2043 | GTACACTGCATCTTCACAGCG |
| 3 | *Escherichia coli* | *rrlB* position - 2333 | TGCCATTGCACTAACCTCCTG |
| 4 | *Escherichia coli* | *rrlB* position - 1854 | ATTGATGGGGTTAGCCGCAAGG |
| 5 | *Escherichia coli* | *rrlB* position - 2814 | TTCCTTCAGGACTCTCAAGGAG |
| 6 | *Escherichia coli* | *rrlB* position - 1714 | TGTAGGTGAGGTCCCTCGCGG |
| 7 | *Escherichia coli* | *rrlB* position - 2814 | TTCCTTCAGGACCCTTAAAGGG |
| 8 | *Escherichia coli* | *rrlB* position - 1711 | ATATGTAGGTGAAGCGACTTG |
| 9 | *Escherichia coli* | *rrlB* position - 2214 | GATCACGGGTCCACGTTAGAAC |
| 10 | *Escherichia coli* | *rrlB* position - 1209 | TGTGAAGGTGTACTGTGAGGT |
