# Supplementary material for: Mitochondrial 16S rRNA Is Methylated by tRNA Methyltransferase TRMT61B in All Vertebrates
Source: PLoS Biol. 2016 Sep 15;14(9):e1002557. doi: 10.1371/journal.pbio.1002557 (PMC5025228; doi:10.1371/journal.pbio.1002557)
Supplement: S4 Table — (DOCX) [file pbio.1002557.s012.docx]

**Supplementary Table 4**

| **Method** | **Fragment** | **Primers/ RFLP enzyme** | **Reaction mix** | **conditions** |
| --- | --- | --- | --- | --- |
| PCR | rrl genes | Primer 1 and 2 – fragment one *E.coli*.  Primer 1 and 3 – fragment two *E.coli*. | 5 pmol of forward and reverse primers, 1.25 units of Taq polymerase (Bio-Lab), 1 x reaction buffer (Bio-Lab), 2 mM MgCl2 and 0.2mM dNTP mix. 1 µl of DNA template was used (DNA template was prepared by diluting 2 µl of saturated bacteria media into 100 µl double distilled water (DDW). DDW was added to a final 20 µl reaction volume. | 94°C for 5 minutes, followed by 35 cycles including denaturation (94°C, 30 sec), annealing (69°C, 30 sec) and elongation (72°C, 10 sec for fragment one, and 72°C, 25 sec for fragment two). The cycles were followed by a final extension step (72°C, 7 min). The reaction was concluded at 10^o^C, and stored at -20^o^C until usage. |
| RFLP | 1 | MlucI | 1 µl (10 units) of MlucI, 2 µl of “CutSmart” buffer mix (10X), 10 µl of PCR reaction; 7 µl of DDW were added to a total volume of 20 µl. | 37°C for 30 minutes, followed by an inactivation step of 10 minutes in 80°C |
| PCR | rrl genes | Primer 4 and 5 – *rrlA E.coli.*  Primer 6 and 7 – *rrlB* & *rrlG* (exact sequence) *E.coli*.  Primer 8 and 9 – *rrlC E.coli*.  Primer 8 and 7 – *rrlD E.coli*.  Primer 6 and 5 – *rrlE E.coli.*  Primer 10 and 5 – *rrlH E.coli*. | 5 pmol of forward and reverse primers, 1.25 units of Taq polymerase (Bio-Lab), 1 x reaction buffer (Bio-Lab), 2 mM MgCl2 and 0.2mM dNTP mix. 1 µl of DNA template was used (DNA template was prepared by diluting 2 µl of saturated bacteria media into 100 µl double distilled water (DDW). DDW was added to a final 20 µl reaction volume. | 94°C for 5 minutes, followed by 35 cycles including denaturation (94°C, 30 sec), annealing (66.5°C for *rrlA* and *rrlH*, 62°C for *rrlB* & *rrlG*, 70°C for *rrlC*, 60°C for *rrlD*, 65°C for *rrlE*, all for 30 sec) and elongation (72°C, 90 sec). The cycles were followed by a final extension step (72°C, 7 min). |
